# The Structure of an NDR/LATS Kinase–Mob Complex Reveals a Novel Kinase–Coactivator System and Substrate Docking Mechanism

**DOI:** 10.1371/journal.pbio.1002146

**Published:** 2015-05-12

**Authors:** Gergő Gógl, Kyle D. Schneider, Brian J. Yeh, Nashida Alam, Alex N. Nguyen Ba, Alan M. Moses, Csaba Hetényi, Attila Reményi, Eric L. Weiss

**Affiliations:** 1 Lendület Protein Interaction Group, Institute of Enzymology, Research Centre for Natural Sciences, Hungarian Academy of Sciences, Budapest, Hungary; 2 Department of Biochemistry, Eötvös Loránd University, Budapest, Hungary; 3 Department of Molecular Biosciences, Northwestern University, Evanston, Illinois, United States of America; 4 Department of Cell and Systems Biology, University of Toronto, Toronto, Ontario, Canada; 5 MTA-ELTE Molecular Biophysics Research Group, Eötvös Loránd University, Hungarian Academy of Sciences, Budapest, Hungary; Mount Sinai Hospital, CANADA

## Abstract

Eukaryotic cells commonly use protein kinases in signaling systems that relay information and control a wide range of processes. These enzymes have a fundamentally similar structure, but achieve functional diversity through variable regions that determine how the catalytic core is activated and recruited to phosphorylation targets. “Hippo” pathways are ancient protein kinase signaling systems that control cell proliferation and morphogenesis; the NDR/LATS family protein kinases, which associate with “Mob” coactivator proteins, are central but incompletely understood components of these pathways. Here we describe the crystal structure of budding yeast Cbk1–Mob2, to our knowledge the first of an NDR/LATS kinase–Mob complex. It shows a novel coactivator-organized activation region that may be unique to NDR/LATS kinases, in which a key regulatory motif apparently shifts from an inactive binding mode to an active one upon phosphorylation. We also provide a structural basis for a substrate docking mechanism previously unknown in AGC family kinases, and show that docking interaction provides robustness to Cbk1’s regulation of its two known in vivo substrates. Co-evolution of docking motifs and phosphorylation consensus sites strongly indicates that a protein is an in vivo regulatory target of this hippo pathway, and predicts a new group of high-confidence Cbk1 substrates that function at sites of cytokinesis and cell growth. Moreover, docking peptides arise in unstructured regions of proteins that are probably already kinase substrates, suggesting a broad sequential model for adaptive acquisition of kinase docking in rapidly evolving intrinsically disordered polypeptides.

## Abstract 2

The structure of an ancient signaling complex in the Hippo pathway (an NDR/LATS family kinase) reveals a distinctive activation switch, and shows how this regulator recognizes short and rapidly evolving protein motifs.

## Author Summary

The core organization of systems that relay information inside cells is preserved across vast evolutionary distances. Thus, detailed characterization of these systems’ crucial modules can provide insight into the emergence and adaptation of signaling pathways, and illuminate broadly relevant mechanisms that control cells’ diverse processes. In this paper we describe the first three-dimensional structure of a protein kinase–coactivator complex from budding yeast that is a key component of “hippo” signaling pathways, which direct cell proliferation, fate, and architecture in a wide range of eukaryotes. We show that this kinase–coactivator complex is a dynamic switch controlled by binding events distant from its active site, and that the kinase recognizes specific short motifs in disordered regions of target proteins by a previously unknown mechanism. This substrate docking interaction provides in vivo robustness to the kinase’s regulation of its known targets, and identifies likely new substrates that expand our view of this hippo pathway’s role in cell division. Moreover, during the course of evolution, the short motif that interacts with the kinase’s docking surface appears in rapidly changing intrinsically disordered regions of a number of proteins that are probably already in vivo substrates. Thus, our findings support the idea that proteins evolve more robust functional links to the signaling networks that control them by acquiring short peptide motifs that interface with key conserved signaling modules.

## Introduction

**Fig 1:**
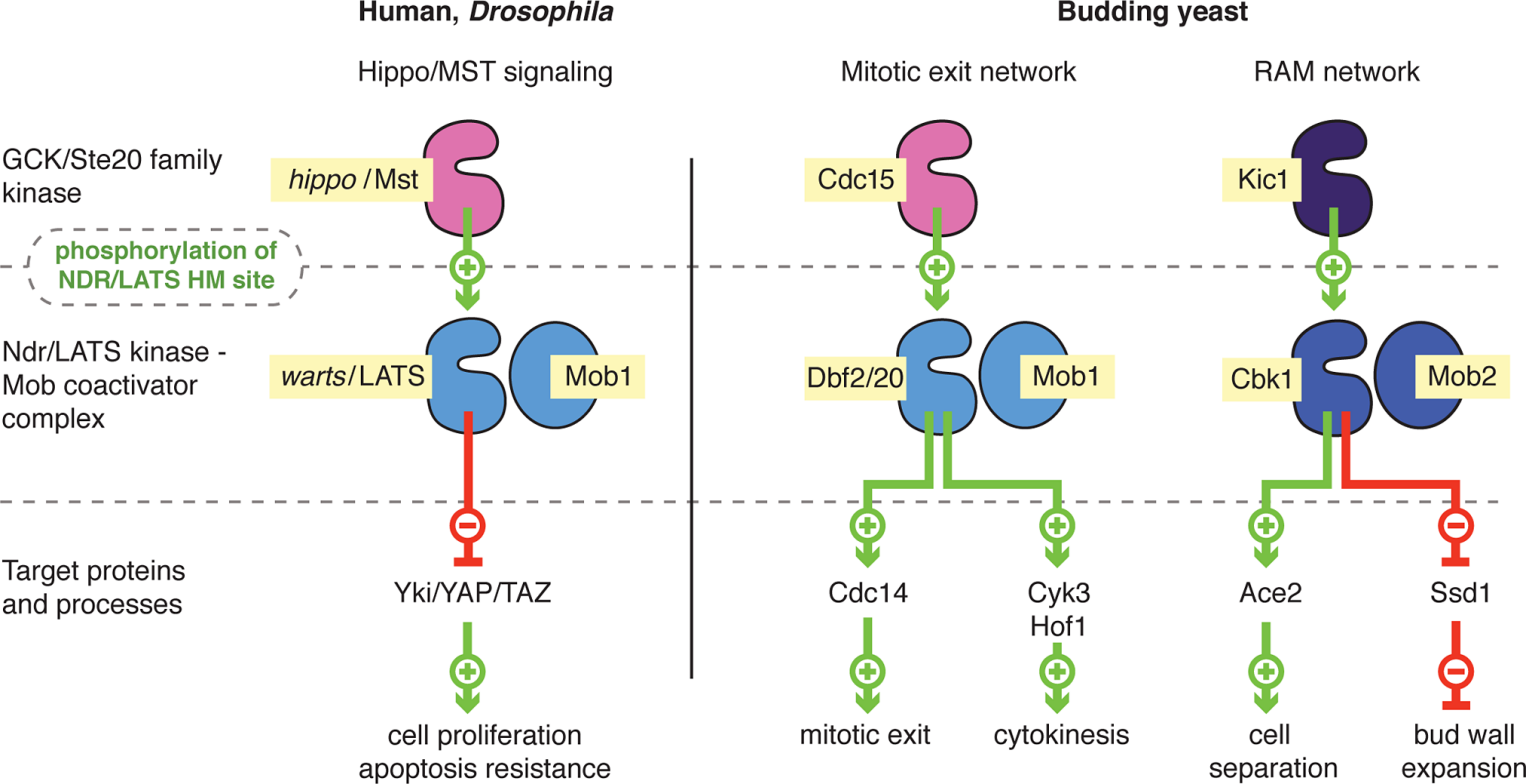
Role of the NDR/LATS kinase–Mob complex in hippo signaling pathways. Left: A metazoan hippo pathway in which a LATS–Mob1 complex is activated by MST/hippo kinases and inhibits the Yki/YAP transcriptional coactivator, based on research from *Drosophila* and mammalian cells. Right: Budding yeast have two distinct hippo pathways, the mitotic exit network (MEN), in which the LATS-related Dbf2 or Dbf20 kinase in complex with Mob1 controls mitotic exit and cytokinesis, and the RAM network, in which the NDR-related Cbk1 kinase in complex with Mob2 controls cell separation and morphogenesis.

“Hippo” signaling pathways control diverse aspects of cell proliferation, survival, and morphogenesis in eukaryotes. The core organization of these networks is conserved over a billion years of evolution, with related forms described in animals and fungi [1–3]. In these systems, MST/hippo kinases activate NDR (nuclear Dbf2-related) or LATS (large tumor suppressor) kinases (Fig 1), which are closely related members of the large AGC family of protein kinases. The NDR/LATS kinases bind to highly conserved Mob coactivators, forming a regulatory complex that controls a diverse set of in vivo effector proteins.

In animals, a form of hippo signaling inhibits cell proliferation and controls tissue architecture [4,5]. In humans and *Drosophila*, LATS kinases in complex with Mob1 family proteins phosphorylate YAP/Yki family transcriptional coactivators, which promote cell division and resistance to apoptosis (Fig 1, left). This sequesters YAP/Yki proteins in the cytoplasm, blocking their activity and thus suppressing cell proliferation and increasing sensitivity to programmed cell death. Notably, activation of metazoan LATS by MST/hippo kinases appears to depend on the kinases’ recruitment to a scaffold protein, and is linked to engagement with cytoskeletal elements and structures at the cell cortex [6]. *Drosophila* and human cells also use another form of hippo signaling, in which MST/hippo activates NDR kinases that form complexes with a different Mob coactivator [3,7]. These pathways control morphogenesis of cell extensions and participate in cell proliferation control, but are much more dimly understood. In *Drosophila*, this “NDR branch” of hippo signaling is important both for proper organization of actin-rich cell extensions and organization of sensory neuron dendrites [8,9]. In mammalian cells, NDR kinases appear to help drive the transition through the G1 restriction point [10].

Hippo pathways are strongly associated with cell proliferation in the budding yeast *Saccharomyces cerevisiae*. One such system called the mitotic exit network (MEN) is centrally important for the transition from mitosis to G1 [11] (Fig 1, right). In this pathway, the yeast hippo-like kinase Cdc15 activates the LATS-related kinases Dbf2 and Dbf20, which bind the Mob1 coactivator protein. The MEN controls key cytokinetic processes and helps flood the cytosol with the phosphatase Cdc14, reversing phosphorylation performed by mitotic CDK [12,13]. MEN activation involves colocalization of Cdc15 and the Dbf2/20–Mob1 complex to the Nud1 scaffold protein at the yeast spindle poles; this recruitment is closely coordinated with the onset of anaphase [12,14].

Budding yeast cells also use an NDR branch of hippo signaling, the RAM network, during the M to G1 transition (Fig 1, right) to initiate the final event of cell division: destruction of an extracellular septum that forms between mother and daughter cells during cytokinesis [15]. In the RAM network, the hippo-like kinase Kic1 activates the NDR-related kinase Cbk1, which forms a complex with the coactivator protein Mob2 [16,17]. Cbk1–Mob2 directly drives nuclear accumulation of the transcription factor Ace2, which turns on expression of septum-destroying hydrolases [18,19]. Intriguingly, Ace2 strongly localizes only to nuclei of newly born daughter cells [20,21]. This produces a sharp expression peak of mother/daughter separation genes that happens only once in a cell’s lifetime, when it is newly born. The RAM network is also required for normal growth and morphogenesis of proliferating and mating cells. While the mechanisms of this regulation are incompletely understood, it partly involves Cbk1’s inhibitory phosphorylation of the RNA-binding protein Ssd1. This allows translation of secreted enzymes that open up the cell wall lattice, allowing its expansion and remodeling in actively growing cell regions [20–23].

NDR/LATS kinase–Mob coactivator modules are centrally important in all known hippo pathways. The catalytic domains and flanking regions of these kinases are extraordinarily well conserved (S1 Fig), as are Mob family proteins [24]. Thus, the NDR/LATS–Mob complex probably has distinctive biochemical characteristics that are specifically adapted to the molecular organization and output requirements of hippo signaling systems in a wide range of eukaryotes. However, these complexes are poorly understood at a structural and mechanistic level. For example, the role of Mob coactivator binding is not understood, and it is uncertain how MST/hippo kinase activation of NDR/LATS kinase–Mob modules works at a biochemical level. For reasons that are unclear, this activation appears to be extremely inefficient in the absence of scaffolding proteins that bring the pathway components together [6,12,14,16,25]. Moreover, relatively few NDR/LATS phosphorylation targets are known, and understanding how these complexes select substrates could identify new ways in which they regulate cellular processes. Furthermore, in addition to illuminating the biology of this ancient pathway, a deep mechanistic understanding of this complex could suggest ways conserved signaling systems change and adapt to control diverse functions as organisms evolve.

Here we describe the crystal structure of the yeast Cbk1–Mob2 complex, providing what is to our knowledge the first structural template for an NDR/LATS kinase–Mob coactivator complex. Combined with molecular dynamics (MD) simulation, this analysis indicates how members of this structurally unexplored kinase group are activated. We find that Mob protein association creates a novel binding pocket that participates in the formation of the active state of NDR/LATS kinases after they are phosphorylated by MST/hippo kinases. This structure also allows us to define how Cbk1’s kinase domain associates with a short peptide motif in its known in vivo phosphorylation targets, a form of substrate docking not previously seen in the broader AGC family of protein kinases. We find that substrate docking confers robustness to Cbk1’s regulation of these proteins. Furthermore, conservation of the short peptide that docks to the Cbk1 kinase domain identifies known targets and strongly predicts an expanded set of kinase substrates, most of which are expected to be involved in cell morphogenesis and cytokinesis. Our analysis also indicates that Cbk1–Mob2 regulatory targets first acquired NDR/LATS phosphorylation consensus sites and subsequently evolved docking motifs in unstructured protein regions, supporting a sequential model for the evolution of docking interactions in kinase–substrate networks.

## Results

### The Structure of Cbk1–Mob2 Reveals a Novel Kinase–Coactivator Complex

Mechanistic insight into the regulation and protein–protein interactions of NDR/LATS kinase–Mob coactivator complexes is limited by absence of crucial information about this important regulatory module’s three-dimensional structure. The budding yeast NDR/LATS kinase Cbk1 and the coactivator protein Mob2 contain characteristic sequence features and regulatory inputs that are preserved across evolution, and have a number of well-characterized in vivo functions. The complex formed by these proteins is therefore a suitable model for Mob coactivator binding, kinase activation, and substrate phosphorylation. To provide the first structural template for NDR/LATS kinase–Mob coactivator complexes, we determined the structure of yeast Cbk1 bound to Mob2 in three different crystal forms (S2 Fig; S1 Table).

We used two variants of catalytically inactive Cbk1(D475A) for crystallography. These differ at position 743, a highly conserved threonine and the phosphorylation site through which MST/hippo kinases (in this case, Kic1) activate NDR/LATS kinases [1,16,26,27]. This phosphorylation site is within a C-terminal catalytic domain extension, and is part of a regulatory hydrophobic motif (HM) that is commonly present in AGC group kinases [28,29]. In addition to Cbk1 intact in its HM region, we used a variant in which threonine 743 was replaced by glutamic acid, an activating substitution that is at least partially phosphomimetic [30,31]. Kic1 phosphorylates this threonine extremely inefficiently in vitro [16], making production of Cbk1 with phosphothreonine at position 743 unfeasible in quantities suitable for biochemical and structural analysis. For clarity, we refer to the HM in which threonine 743 is not phosphorylated as “HM-T,” the glutamic acid substitution at this site as “HM-E,” and the form in which threonine 743 is phosphorylated as “HM-P.”

We collected crystallographic data on one Cbk1(HM-T)–Mob2 complex that diffracted to 3.3 Å, with one Cbk1–Mob2 complex in the asymmetric unit. We also solved the structure of Cbk1(HM-E)–Mob2 in a crystal form that diffracted to 3.6 Å, containing two Cbk1(HM-E)–Mob2 complexes in the asymmetric unit related by noncrystallographic symmetry (NCS). Another crystal form of the Cbk1(HM-E)–Mob2 complex diffracted to 4.5 Å, with one complex in the asymmetric unit.

**Fig 2:**
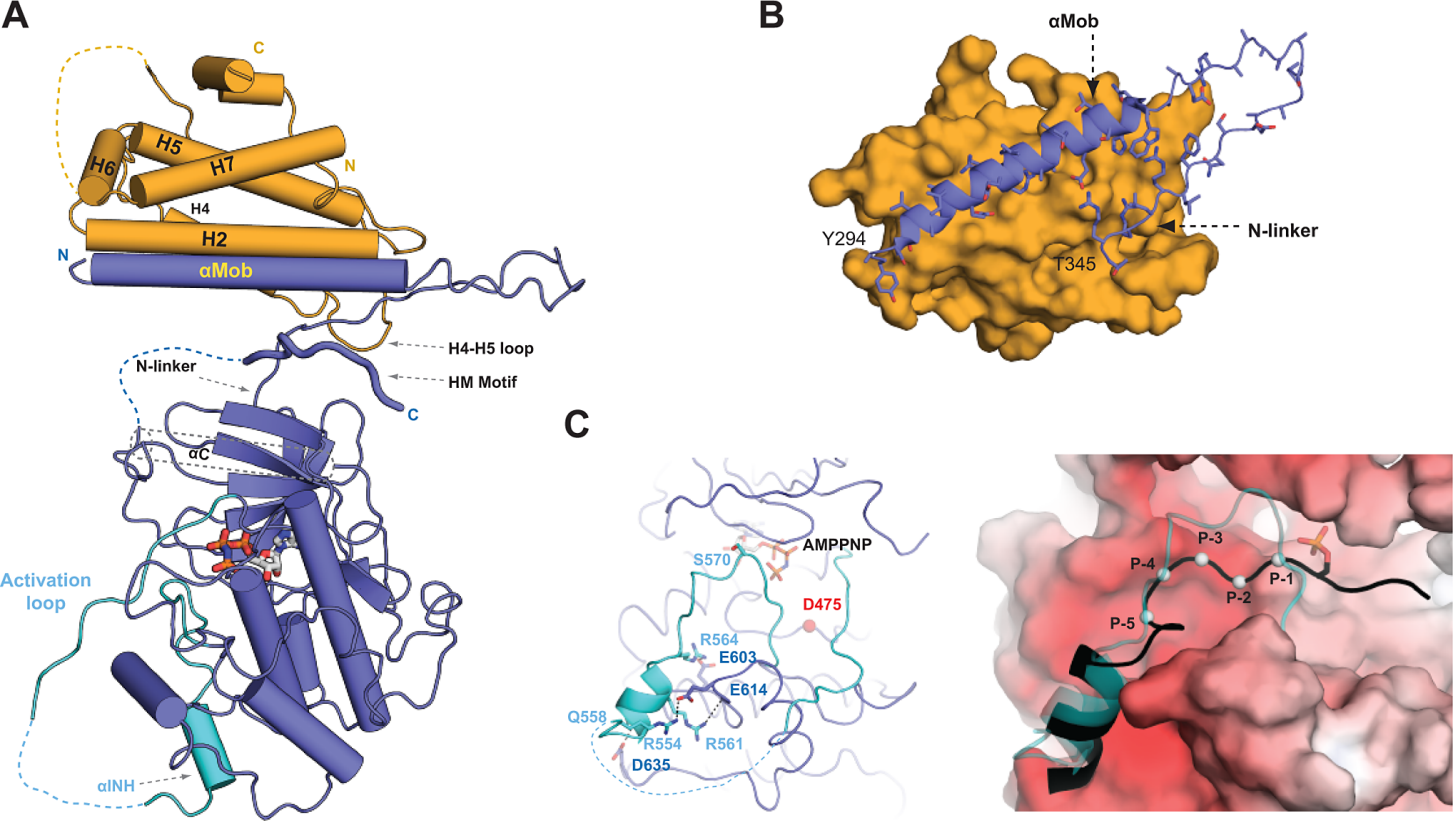
Structure of the Cbk1–Mob2 complex. (A) An overview of the Cbk1–Mob2 complex. Mob2 (orange) binds to the N-terminal region of Cbk1 (blue) through a large surface formed by the H2 and H7 helix and the H4–H5 loop. “N” and “C” denote the N- and C-terminal ends of the protein constructs. Flexible regions that could not be located in the electron density maps are shown with dashed lines. HM is the AGC kinase HM, and “αINH” denotes the inhibitory alpha helix that is part of the activation loop (shown in cyan). (B) The N-terminal region of the Cbk1 forms a bipartite Mob2-binding surface comprised of a long αMob helix and a highly conserved arginine rich N-linker region. (C) An activation loop segment (cyan) of Cbk1 containing the major autoregulatory site (S570) blocks access to the active site (D475) (left panel). An inhibitory helix (αINH in [A]) is anchored in the substrate-binding groove by several salt bridges (shown with black dotted lines). The substrate binds in the negatively charged “open” binding pocket that replaces αINH (shown in cyan), as seen in the state with the bound αINH (right panel). The model for the Cbk1–substrate complex was generated by superimposing the crystallographic model of the “open” state of Cbk1 with PKA binding to a phosphorylated substrate (Protein Data Bank [PDB] ID: 1JLU). The black cartoon with gray spheres (from P-1 to P-5 Cα positions) shows the PKA substrate superimposed in the Cbk1 substrate-binding pocket, while the activation loop of Cbk1, which displays the characteristics of a pseudo-substrate region, is shown with the semitransparent cartoon representation in cyan. The surface of Cbk1 is colored according to its electrostatic potential, where red indicates negatively charged surface. The nucleotide cofactor (AMPPNP) is shown in stick representation.

The domain arrangements for the kinase and its coactivator are the same in all final crystallographic models (S2A Fig). In each structure, Mob2 binds to the conserved N-terminal extension of the Cbk1 AGC kinase domain, a region previously identified as the site by which NDR kinases bind Mob proteins [32] (Fig 2A). A portion of Cbk1’s N-terminal extension forms a long helix (termed “αMob”), which is a major site of contact with Mob2 (Fig 2B). The αMob helix is followed by a sharp turn and then a short segment, here termed the “N-linker,” that connects to the core kinase catalytic domain. Mob2 also interacts with the N-linker, which together with the αMOB helix forms a >1,100 Å^2^ bipartite interaction surface between the kinase and its coactivator. All structures of the Cbk1–Mob2 complex reveal a deep cleft between the kinase N-terminal lobe and the Mob-binding interface formed by αMOB and the N-linker. The crystal structure of human and budding yeast Mob1 proteins have been solved [33,34], and the fundamental structure of Mob2 in complex with Cbk1 is essentially similar.

In addition to their Mob-binding N-terminal kinase domain extensions, NDR/LATS kinases typically have notably elongated activation loop segments [35]. Superimposing our Cbk1 structures onto those of ROCK and PKA [36,37] shows that both of these Cbk1 regions adopt distinctive conformations (S2B Fig). Cbk1’s activation loop forms multiple different structures: the higher resolution Cbk1(HM-E)–Mob2 crystallographic model captures two of these conformations displayed by the two NCS-related models (Fig 2C). In one form, structured parts of the activation loop occupy the substrate-binding pocket and thus occlude access to the catalytic site, similar to the structure of PKA complexed with an alpha helical peptide inhibitor (S2C Fig) [35,38]. In the other complex, the activation loop is not present in the substrate-binding region. Cbk1(HM-T)–Mob2 complex crystallized in only one of these states, and its substrate-binding pocket was blocked. We suggest that this is an auto-inhibited form of the kinase, while the other state seen in one of the two complexes present in Cbk1(HM-E) crystals may be competent to bind substrates containing basic amino acids (Fig 2C, right). Although crystal packing interactions around the kinase activation loop are clearly different for the two complexes of the Cbk1(HM-E)–Mob2 model, it is likely that the extended activation loop of NDR/LATS kinases switches them between an inhibited and open state, with phosphorylation of the conserved autoregulatory site S570 (near AMPPNP in Fig 2C, left) alleviating the pseudo-substrate-based inhibition exerted by the inhibitory helix (αINH). The activation loop displays dramatically different organization between each crystal form as well as between NCS-related molecules of the same crystal (S2D Fig), and none of these contain an intact catalytic center that can phosphorylate the autoregulatory site, preventing further speculation. Thus, further work will be necessary to decipher the regulatory role of activation loop inhibition and alleviation by autophosphorylation, as well as to identify the structural elements important for substrate binding.

In many AGC kinases, the C-terminal HM region associates with the catalytic domain’s N-terminal lobe in a conformation that stabilizes the important C helix, promoting enzyme activation. In contrast, the Cbk1–Mob2 complex structures we solved exhibit a novel organization of the HM region and activating phosphorylation site. In all three structures, the Cbk1 HM peptide is wedged into a binding slot in the deep cleft formed by the kinase’s αMob helix, N-linker region, and N-terminal catalytic domain lobe (Figs 2A and S2E and S2F). Mob2 does not contact the HM region directly, but rather holds the HM-binding cleft formed by Cbk1’s N-terminal extension open. In this location the HM peptide probably cannot productively structure the C helix; consistent with this, the C helix region is disordered in all structures we solved (Figs 2A and S2A and 3A).

### Cbk1 HM Phosphorylation Suggests an Activation Mechanism Unique to NDR/LATS Kinases

How does the conserved phosphorylation site in the NDR/LATS HM region regulate these kinases? While replacement of the phosphorylated threonine with glutamic acid at this site increases Cbk1’s activity in vitro and in vivo, it is uncertain how closely this mutation mimics the biochemical effect of phosphorylation. In our crystal structures, unphosphorylated wild-type (WT) HM region (HM-T) and HM region with glutamic acid substitution (HM-E) associate with the Mob2-supported binding site in a similar manner. If these closely related structures capture the HM region in its kinase-activating conformation, then phosphorylation of the HM site might be expected to strongly increase the HM region’s affinity for Cbk1. We measured the affinity of HM peptides added in trans to Cbk1–Mob2 complex in which Cbk1’s native HM region was truncated. Phosphorylated peptide (HM-P) bound with an affinity of approximately 6 μM, while the dephosphorylated peptide (HM-T) bound with an affinity of about 33 μM (S3 Fig). The HM–Cbk1 interaction is intramolecular, putting the HM peptide’s effective local concentration in the millimolar range. The modest difference in affinity of HM and HM-P peptides argues that HM site phosphorylation does not achieve its regulatory effect by enhancing the region’s ability to bind at a key regulatory site on the kinase.

**Fig 3:**
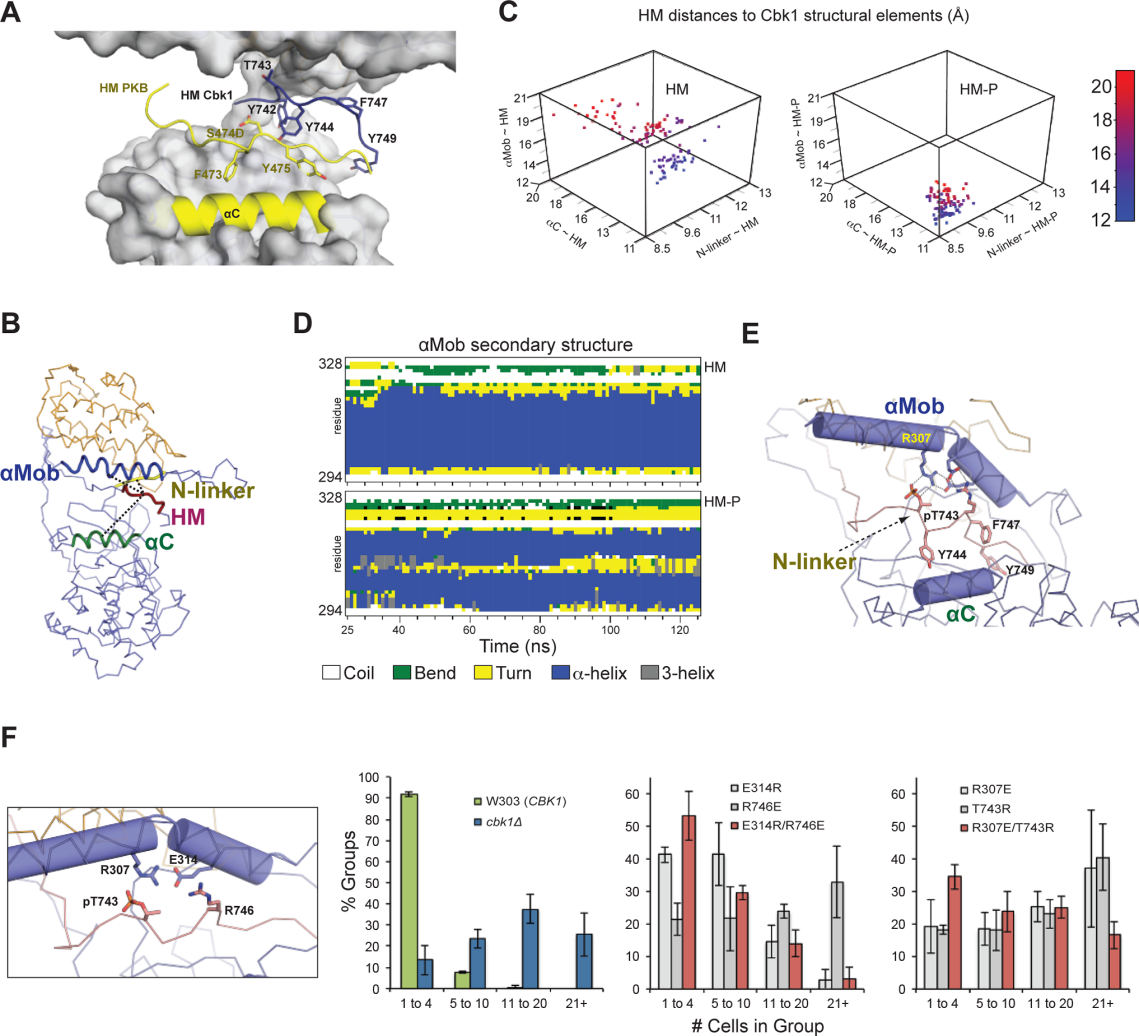
Molecular dynamics simulation indicates phosphorylation-induced rearrangement of the NDR/LATS kinase HM-binding slot to favor enzyme activation. (A) Comparison of the Cbk1 HM with the HM of PKB. The panel shows the Cbk1–Mob2 complex superimposed with PKB, where the αC helix and the HM of the superimposed PKB are shown in yellow (PDB ID: 1O6K). Side chains of important HM residues are shown with sticks. Superimposition of PKB and Cbk1–Mob2 reveals that the main chains of the HM motifs are >7 Å apart. The Cbk1 HM motif is shifted upwards to bind to the upper part of the HM-binding slot, while the PKB HM binds close to the N-terminal PKB kinase lobe. (B) Cbk1–Mob2 complexes in which T743 in the C-terminal HM is either dephosphorylated (HM-T) or phosphorylated (HM-P) were subjected to 125-ns MD simulations. Here we represent indicated regions of Cbk1 as centers of mass of the following: HM, amino acids 742–749; N-linker, amino acids 341–346; αC, amino acids 393–406; αMob, amino acids 297–317. We started distances of both HM forms to the N-linker region, αC, and αMob at [8.7 Å; 18.0 Å; 14.9 Å], corresponding to the conformational state captured in the Cbk1–Mob2 complex crystal structure, and recalculated the distances between HM forms, N-linker, αC, and αMob after each simulation step. (C) Three-dimensional scatter plots show 100 ns of the simulations, with each dot indicating N-linker–HM, αC–HM, and αMob–HM distances (*x-*, *y-*, and *z*-axes) for each nanosecond. For clarity, dots are colored from blue to red according to their αMob–HM distances (*z*-axis). The complex with HM-P exhibits shorter distances and smaller changes in position, indicating that T743 phosphorylation compresses the HM-binding slot and constrains the HM region association. This occurs only in Cbk1 bound to Mob coactivator (see S5 Fig). (D) HM phosphorylation promotes αMob bending and compresses the HM binding slot. Secondary structure analysis of αMob illustrates that this region remains helical (blue) when HM-T is bound, but acquires a short turn (yellow) when HM-P is bound. (E) An enlarged view of the final MD-simulated Cbk1(HM-P)–Mob2 complex indicates the HM-P’s interactions that bend αMob, as well as van der Waals contacts with αC. (F) Ionic interactions suggested by the MD model (E314/R746 and R307/pT743) (left) were tested by charge swapping and analysis of cell separation. Single mutants (grey) displayed larger group sizes, indicating defective RAM network function, but were partially recovered when both charged sites were swapped (red). Data for (C) and (F) can be found in S1 Data.

Since it was not possible to produce Cbk1 with phosphorylated HM (HM-P) for biochemical and crystallographic characterization, we performed MD simulations using the Cbk1–Mob2 structural template to develop a plausible model for the function of HM phosphorylation. Since the αC helix of Cbk1 was not resolved, we used homology modeling to build a folded αC based on the structure of activated PKB [39]. We then conducted 125-ns MD simulations using models with either HM-T or HM-P (Figs 3A and S4). To estimate the mobility and position of Cbk1’s HM region in these two models, we calculated the distance between the centers of mass of peptide segments encompassing Cbk1’s N-linker, αMob, αC, and HM regions (Fig 3B).

This analysis clearly predicts that phosphorylation of Cbk1’s HM site significantly affects both the HM region’s mobility and the structure of the site it associates with in the Cbk1–Mob2 complex. As noted in Fig 3C, the mobility of HM-P is significantly lower than the mobility of HM-T, and HM-P and the αC, N-linker, and αMob segments are closer to each other (Fig 3C). Cbk1’s N-linker region is compressed as the HM-P fragment binds in a region analogous to the PIF pocket and directly interacts with αMob, producing a bend in αMob that does not occur in the HM-T model (Fig 3D and 3E). The simulations predict that phosphorylated T743 compresses the N-linker pocket by making salt bridge contacts with αMob. Notably, two residues (R746 and F747) make direct contacts with αMob and αC (Fig 3E). These are invariant in the NDR/LATS lineage, but are not conserved in AGC kinases that lack Mob cofactors. Despite αMob reorganization, MD simulation indicates that the Mob interaction interface probably remains largely unchanged when HM-P interacts (S4A Fig). Moreover, αMob’s interaction with HM-P occurs only when the Mob coactivator is present (S4B Fig). Similar MD analysis of the Cbk1(HM-E)–Mob2 structure indicates that HM-E also has more restrained movement of the HM region within the binding slot than HM-T, but slot compression was significantly less pronounced compared to HM-P (S4C Fig). This suggests that HM-E is only a partial substitute for HM-P.

MD simulation suggests that HM site phosphorylation promotes formation of multiple electrostatic salt bridge interactions. We therefore conducted in vivo charge swap experiments with two of these putative salt bridges, R307/pT743 and E314/R746 to test the validity of the simulations. As outlined in Fig 1, Cbk1 is required for mother/daughter separation; pathway output can be measured by counting the number of cells present in physically associated groups in liquid culture [19]. WT budding yeast cells separate well (about 90% of cell groups contain one to four cells), while *cbk1*Δ strains fail to separate: they grow in large clumps with about 80% of groups containing five or more cells (Fig 3F). We introduced single and double mutations of the putative HM salt bridge interactions (R307E/T743R and E314R/R746E) and measured cell group sizes. Both charge swaps showed recovery in the double mutant relative to the single mutants, with E314R/R746E returning to 50% in groups with one to four cells per group and <5% with 21 or more cells per group relative to the R746E single mutant levels of 20% and 30%, respectively. The R307E/T743R mutant also displayed some recovery, with ~35% in groups with one to four cells per group and ~20% in groups of 21 or more. The lower degree of recovery for this latter mutant is consistent with MD simulation, which suggests that pT743 engages in multiple interactions that the R307E/T743R variant would not recapitulate. In summary, our findings support an activation model specific to NDR/LATS kinases that requires Mob cofactor binding, in which HM site phosphorylation induces compression of the N-linker region to produce a PIF-pocket-like binding site.

### A Conserved Substrate Docking Motif Associates with a Specific Site on Cbk1’s Kinase Domain

**Fig 4:**
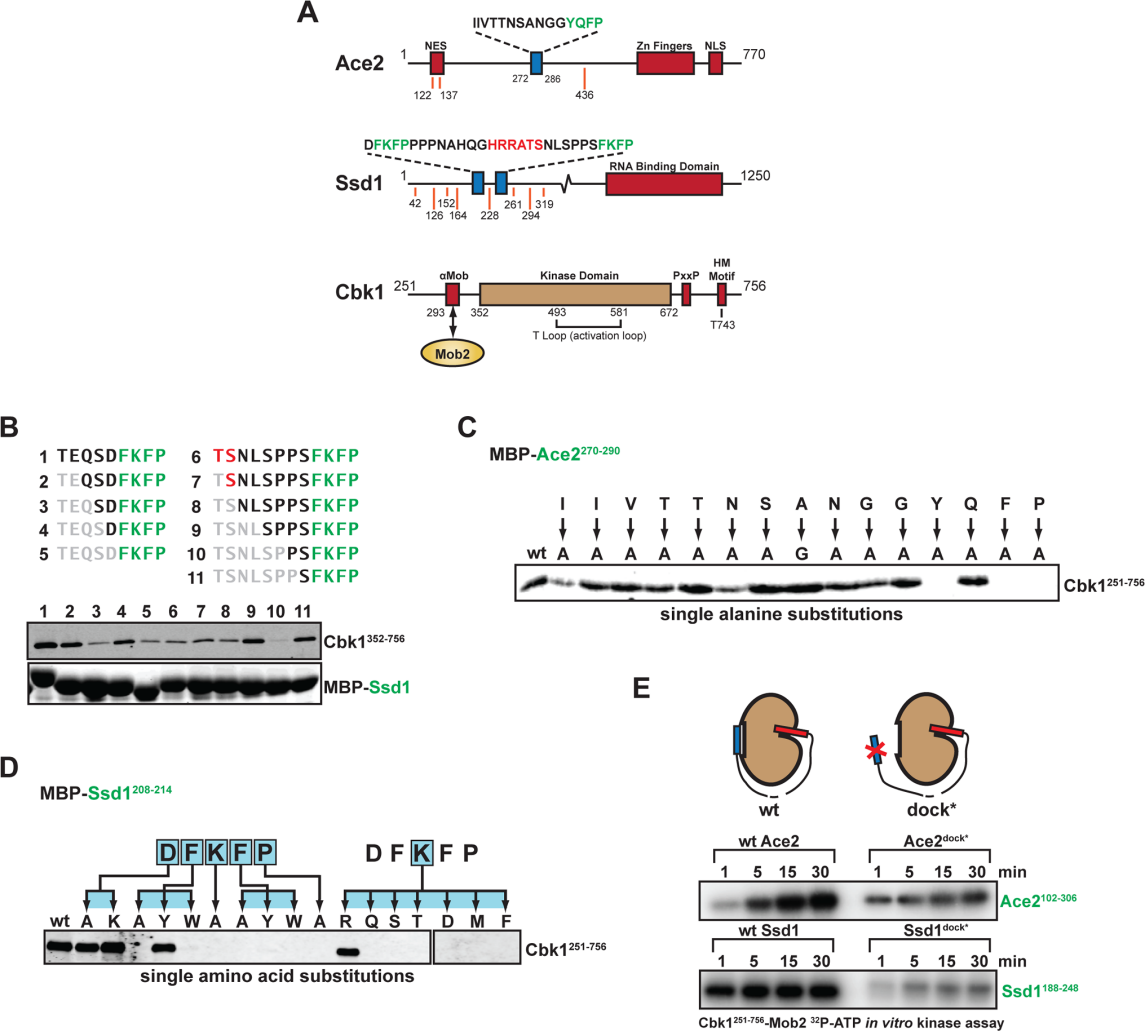
Molecular analysis of Cbk1 docking peptides in Ace2 and Ssd1 highlights importance of a [YF]xFP motif. (A) Protein schematics with points of interest highlighted. Orange lines denote Cbk1 consensus sites, and blue boxes denote docking motifs. (B) Pulldown of Cbk1 kinase domain by truncated Ssd1 N-terminal (1–5) and C-terminal (6–11) docking motifs suggests that only the FKFP motif is required for interaction. (C) Alanine scan of the Ace2^270–290^ docking motif suggests that residues N-terminal to the YQFP motif aid in Cbk1 binding. (D) Mutational analysis of Ssd1 N-terminal docking motif highlights the sequence stringency of the core motif and suggests a consensus docking motif of [YF][KR]FP. (E) Cbk1 in vitro kinase assay with Ace2 or Ssd1 fragments containing a phosphorylation site (HxRxx[ST]) and either a WT docking motif (left) or mutated docking motif (dock*, right). Phosphorylation is enhanced in the presence of the WT docking motif.

We sought to use the crystal structures of Cbk1–Mob2 to better understand how the complex recognizes targets of regulatory phosphorylation in vivo. The sites that Cbk1 and other NDR/LATS kinases phosphorylate provide some specificity: many kinases prefer phospho-acceptor sites with specific amino acids immediately nearby (HxRxx[ST] in the case of Cbk1 and other NDR/LATS kinases) [18,19,40,41]. However, these sequence motifs are generally not sufficient to distinguish in vivo substrates from proteins that contain them by chance. Notably, it has been discovered that some protein kinases bind additional short specificity-enhancing motifs (referred to as docking motifs) in substrate proteins [42,43]. We previously found a conserved short peptide motif ([YF][QK]FP) on the Cbk1 substrates Ace2 and Ssd1 that associates with bacterially expressed Cbk1 in vitro [44] (Fig 4A). As described below, we find that specific features of peptides of this motif associate with a site on Cbk1’s catalytic domain, and that the segments’ presence in substrate proteins significantly enhances phosphorylation by Cbk1 in vitro. We therefore consider them bona fide Cbk1 docking motifs, and refer to them as such [42,43].

We first characterized binding of Ace2 and Ssd1 peptide segments to Cbk1’s catalytic domain and the Cbk1–Mob2 complex in vitro. This showed that a 15-amino-acid peptide from Ace2 with the sequence YQFP at its C-terminal end associates with the Cbk1–Mob2 complex, and that two shorter peptides from Ssd1 containing the motif FKFP also interact with this complex (Figs 4B and S5A and S5E). Ace2 and Ssd1 peptides compete for binding, indicating that they likely associate with the same kinase surface (S5G Fig). Fluorescence polarization (FP) analysis using labeled synthetic peptides shows that the Ace2 peptide binds Cbk1–Mob2 with a ~6 μM K_d_, and a peptide from Ssd1 containing the FKFP motif binds with a ~20 μM K_d_ (S5H Fig). Both the Ace2 and Ssd1 fragments interact with Cbk1 lacking N- and C-terminal extensions, indicating that their association occurs within the catalytic domain (S5D and S5I Fig).

To define the Ace2 and Ssd1 peptide features that mediate association with Cbk1, we systematically introduced amino acid substitutions into the peptides and assessed their binding to the Cbk1 catalytic domain and the Cbk1–Mob2 complex. Substitution of alanine for either of the aromatic amino acids or for the proline in the [YF]xFP motif eliminates in vitro association with Cbk1, demonstrating that this amino acid sequence is critical for the peptides’ interaction with the Cbk1 kinase domain (Fig 4C and 4D). We thus refer to [YF]xFP as the “core motif” required for Cbk1 association.

The [YF]xFP core motif is conserved and critical for Cbk1 binding, but the Ace2 and Ssd1 peptides exhibit distinct preferences for amino acids within it and in the surrounding sequence. Several lines of evidence suggest that Ace2’s Cbk1-binding peptide has a functionally bipartite structure. First, a short region at the N-terminal end of this 15-amino-acid-long peptide is important for maximal association with Cbk1–Mob2 (Fig 4C); the requirement for these N-terminal amino acids is more pronounced in assays performed with the catalytic domain alone (S5B Fig). Second, replacement of amino acids near the center of Ace2’s Cbk1-binding peptide with alanine does not affect interaction with the kinase. Third, the Ace2 peptide contains two consecutive glycine residues N-terminal to the core motif, and variants containing one to four glycines still bind Cbk1, while eliminating these residues abolishes interaction (S5C Fig). Thus, the Ace2 docking peptide probably has two distinct short Cbk1-binding epitopes connected by a flexible linker.

In contrast, Ssd1’s Cbk1-binding peptide requires only the core [YF]xFP motif to associate with the kinase. Phylogenetic analysis, however, indicates that arginine and lysine are conserved in the second position of the motif ([YF]KFP or [YF]RFP), and these basic amino acids are critical for Cbk1 binding in vitro (Fig 4D). Basic amino acids are not present in the core motif of the Ace2 peptide, which has the sequence YQFP. Thus, the extended binding configuration of Ace2’s peptide likely changes the constraints on the sequence of the core motif, relieving the need for basic amino acids in the second position (as seen in the Ssd1 peptides). Intriguingly, the Ssd1’s core FKFP motif does not bind Cbk1’s kinase domain when present in Ace2 peptides with mutated N-terminal regions, indicating that the peptide context in which the core motif is displayed influences its ability to bind the kinase (S5F Fig).

Docking motifs in substrate proteins can dramatically increase their rate of phosphorylation by kinases that bind to them [43,45]. We therefore determined if Cbk1-binding peptides from Ace2 and Ssd1 enhance phosphorylation of these substrates, comparing phosphorylation of Ace2 and Ssd1 protein fragments containing either native Cbk1-binding peptides or mutated sequences. The presence of WT Cbk1-binding segments in Ace2 and Ssd1 fragments significantly increases their in vitro phosphorylation by both the Cbk1–Mob2 complex and the Cbk1 kinase domain alone (Fig 4E).

**Fig 5:**
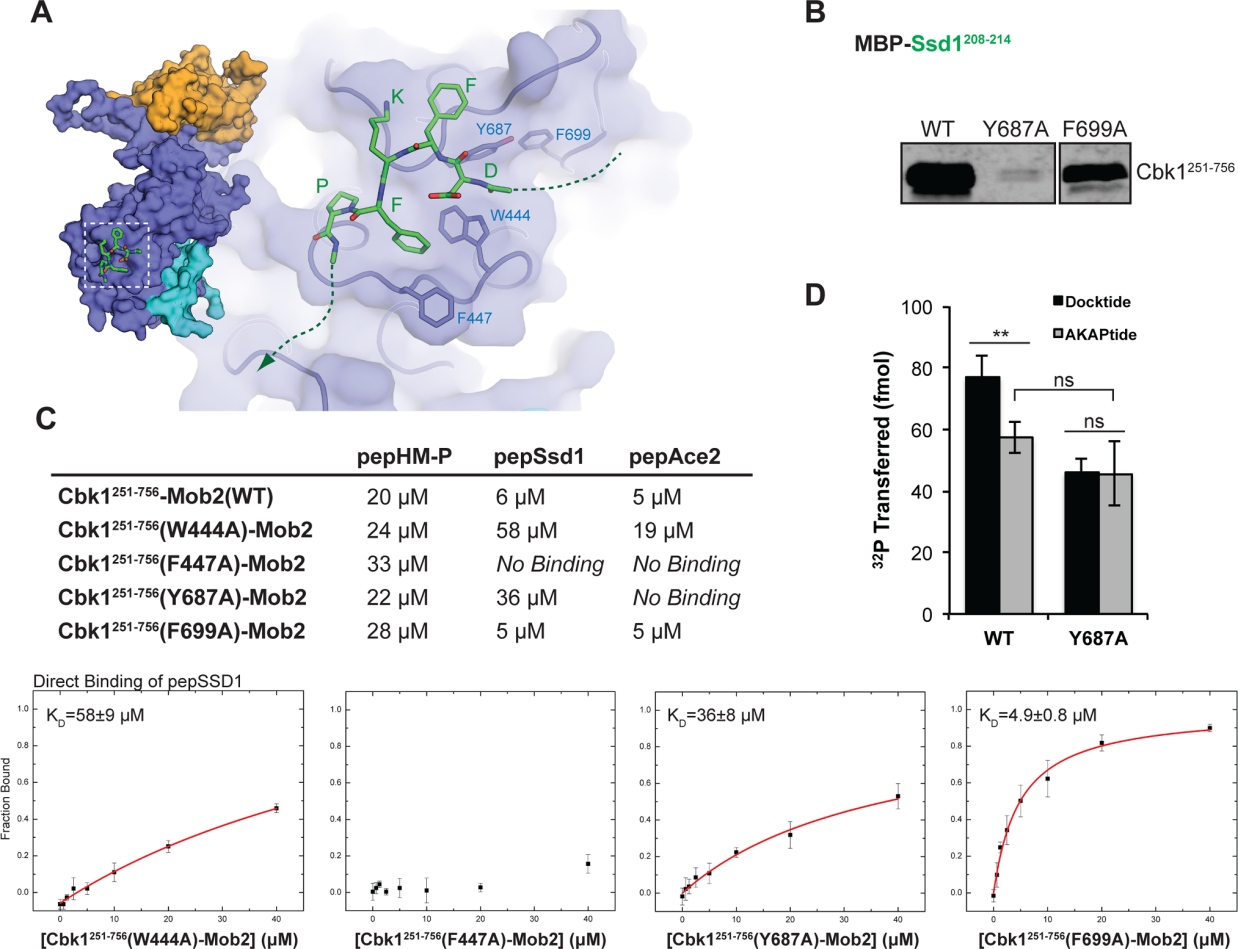
Structural model of the Cbk1–docking peptide complex. (A) AutoDock binding simulation of Ssd1 docking fragment (DFKFP) identifies an interaction surface on Cbk1 and suggests important aromatic interactions with residues W444, F447, and Y687. (B) Y687A but not nearby F699A abrogate Cbk1 pulldown by Ssd1^208–214^ as predicted by the model. (C) FP analysis of putative docking motif binding site residues. The binding of all mutants to pepHM-P was similar to that of WT Cbk1, indicating that the mutants are folded properly (see first column in the table summarizing peptide binding affinities). W444A, F447A, and Y687A were reduced in their binding affinity for Ssd1 and Ace2 docking peptides, while F699A was not. (D) Peptide kinase assay of WT and Y687A mutant of Cbk1^251–756^–Mob2. Peptides are based on Ssd1 (210–229), which contains the N-terminal docking motif (Docktide: FKFP, AKAPtide: AKAP) and a Cbk1 consensus site. The presence of the WT docking motif enhances phosphorylation by WT Cbk1 relative to a peptide containing a mutant docking motif (similar to Fig 4F). Cbk1(Y687A) loses this enhancement when unable to bind WT docking motif. Data is plotted as mean ± standard error of the mean. Student’s unpaired *t*-test: **p < 0.01; ns, not significant. Data for (C) can be found S2 Data, and data for (D) can be found in S3 Data.

Our attempts to use crystallography and crosslinking to understand how Cbk1 interacts with the Ace2 and Ssd1 peptides were not successful. We therefore used unbiased blind docking of the Ssd1 DFKFP peptide to the Cbk1 kinase domain using AutoDock [46]. This computational approach strongly supports docking motif association with a pocket formed by a PxxP region in Cbk1’s C-terminal extension (where Y687 follows the first proline) and a surface loop connecting the D and E helices in the C-terminal kinase lobe (W444, F447) (see S1 Fig). This computationally supported peptide docking site is consistent with the crucial importance of aromatic amino acids in the peptide and predicts that the aromatic amino acids F447, W444, and Y687 in Cbk1 are involved in the interaction (Fig 5A). We tested these predictions by substituting alanine at these sites individually and assessing in vitro binding of docking peptides to the mutant proteins. As predicted, F447A, W444A, and Y687A substitutions abolish or greatly reduce binding of Cbk1–Mob2 to Ace2 and Ssd1 docking peptides. However, HM-P binding in trans stays intact, indicating correct folding and structural integrity of all tested mutants (Fig 5B and 5C).

Cbk1’s PxxP motif is in the C-lobe tether (CLT) region that is conserved in AGC kinases [28]. In other AGC kinases, mutation of the PxxP motif can disorder the CLT, which compromises catalytic activity. We therefore determined if mutation of Cbk1’s PxxP motif has such an effect by measuring the kinase activity of Cbk1(Y687A). This mutation eliminates enhancement of substrate phosphorylation conferred by the presence of an FKFP docking motif, as expected from direct binding experiments, but, importantly, does not compromise basal kinase activity on substrates lacking a functional docking motif (Fig 5D).

### Substrate Docking Confers Robustness to Functional Kinase–Substrate Interactions In Vivo

**Fig 6:**
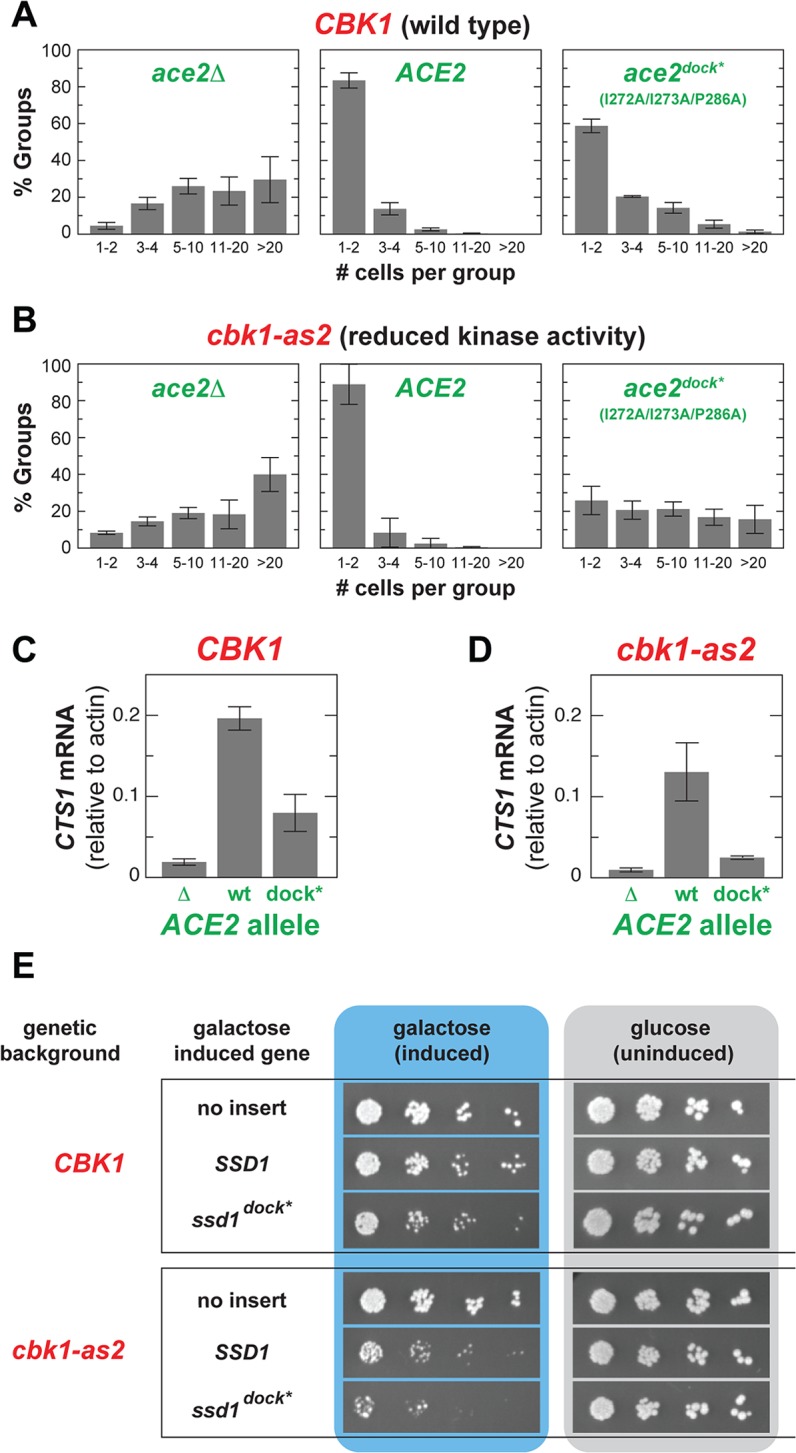
Docking sites increase robustness of Cbk1 control of in vivo substrates. (A) Mutation of the Cbk1 docking motif in Ace2 (*ace2* ^dock*^) confers a modest defect in cell separation in cells with WT *CBK1*. (B) The *ace2* ^dock*^ allele has a marked cell separation defect in cells carrying the *cbk1-as2* allele, which is catalytically weakened. Note that *cbk1-as2* cells exhibit no cell separation defect when the WT *ACE2* allele is present. (C and D) *CBK1* WT cells carrying *ace2* ^dock*^ have a slight reduction in *CTS1* transcript levels (C), while *cbk1-as2* cells carrying *ace2* ^dock*^ exhibit strongly reduced *CTS1* transcription (D). (E) Overexpression of the *ssd1* ^dock*^ allele, carrying mutations that eliminate docking interaction with Cbk1, affects viability of WT cells, and this is far more dramatic in *cbk1-as2* cells. Cells were 10-fold serially diluted from left to right and plated on galactose (inducing) or glucose (repressing) media: reduced colony formation in serial dilutions reflects impaired viability. Data for (A) and (B) can be found in S4 Data, and data for (C) and (D) can be found in S5 Data.

To determine the in vivo function of Cbk1’s docking interactions with its known substrates, we evaluated phenotypic effects of mutations that eliminate the peptides’ in vitro interaction with Cbk1’s kinase domain. We introduced mutations into Ace2’s Cbk1 docking motif that abrogate the peptide’s in vitro interaction with the kinase (an allele termed *ace2* ^dock*^). In cells expressing *ace2* ^dock*^ there was a shift towards larger groups of cells—60% of groups contained 1–2 cells, and 20% of groups had five or more cells—though not nearly to the extent of *ace2*Δ strains (<5% of groups contained 1–2 cells and >95% of groups contained five or more cells per group) (Fig 6A).

These results show that *ace2* ^dock*^ is a partial loss-of-function allele under ideal growth conditions. We therefore hypothesized that the docking interaction enhances robustness of this kinase–substrate interaction, buffering the system against variability in RAM network activity and maintaining constant signaling output. This predicts that docking interaction should be far more important when Cbk1 activity is compromised. We tested this by measuring cell separation in strains expressing the hypomorphic Cbk1-as2 (M429A) mutant protein. This mutant protein, which allows inhibition of Cbk1 by modified ATP analogs, has significantly reduced intrinsic kinase activity in the absence of drug (we performed experiments without drug addition) [21,46]. Notably, the catalytically weakened *cbk1-as2* allele has absolutely no phenotype in cells with WT *ACE2*. However, combining the *cbk1-as2* and *ace2* ^dock*^ alleles dramatically disrupts cell separation: only ~25% of groups contained 1–2 cells, while ~54% contained five or more cells (Fig 6B). We also measured transcription of three Ace2 target genes. Expression of the Ace2-driven genes *CTS1*, *DSE1*, and *SCW11* was reduced in *ace2* ^dock*^ cells, and was nearly absent in *ace2* ^dock*^ *cbk1-as2* cells (Figs 6C and 6D and S6).

Cbk1’s phosphorylation of the mRNA binding protein Ssd1 allows translation of proteins required for cell wall expansion in the growing bud (Fig 1), and loss of RAM network activity is lethal in cells with functional Ssd1. Moreover, overexpression of WT *SSD1* is mildly toxic to WT cells but dramatically deleterious in *cbk1-as2* cells, and overexpression of an Ssd1 variant lacking Cbk1 phosphorylation consensus sites is lethal [23,47]. Thus, the strength of Cbk1’s regulation of Ssd1 can be assessed using viability effects. We compared viability of strains overexpressing either WT *SSD1* or an allele in which Cbk1 docking motifs are mutated (termed *ssd1* ^dock*^). Overexpression of *ssd1* ^dock*^ subtly compromises the viability of WT cells, and this effect is markedly worse in *cbk1-as2* cells (Fig 6E).

Our findings show that docking motifs in Ace2 and Ssd1 stabilize their regulation by Cbk1. This suggests that Cbk1–substrate docking with target proteins in vivo confers signaling system robustness, maintaining pathway output when input signal strength fluctuates or is compromised.

### The Cbk1 Docking Motif Predicts an Expanded System of RAM Network Regulatory Targets

The docking motifs in Ace2 and Ssd1 are conserved in these proteins over hundreds of millions of years of fungal evolution [44], suggesting that the signaling robustness they provide confers a selective advantage. It therefore seems reasonable that this kind of kinase–substrate docking in other Cbk1 regulatory targets might also increase fitness. We therefore evaluated conservation of both the core [YF]xFP Cbk1 docking motif and the distinctive Cbk1 phosphorylation consensus sequence (HxRxx[ST]) in all budding yeast proteins. We used the algorithm ConDens [48], which assigns a conservation score to each instance of a specified short motif based on its preservation relative to surrounding amino acids and its degree of enrichment within a protein region above random expectation. This approach quantifies motif conservation independent of precise sequence alignment. Notably, however, motifs that occur in regions of high background conservation are given a low score.

**Fig 7:**
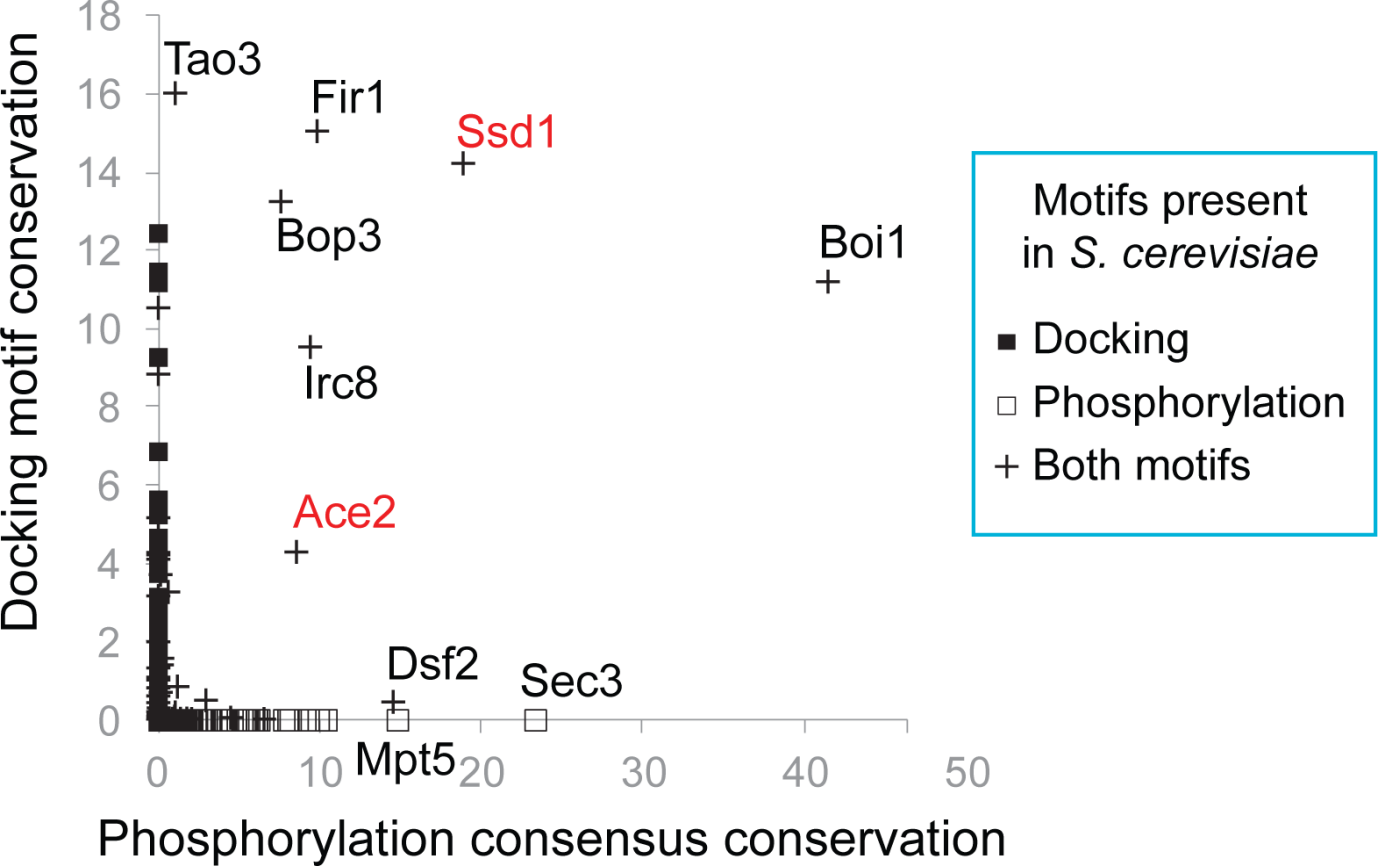
Co-conservation of docking and phosphorylation sites identifies known and likely Cbk1 regulatory targets. Conservation of Cbk1 docking motifs and phosphorylation consensus sites in individual proteins, calculated using ConDens [48]. For each motif type, the most significant score among the matches in each protein is assigned as the score for that protein. The *x*-axis plots conservation scores of [YF]xFP sequences (Cbk1 docking), and the *y*-axis plots conservation scores of Hx[RK]xx[ST] sequences (Cbk1 phosphorylation consensus). The known Cbk1 substrates Ssd1 and Ace2 are highlighted in red. Proteins without docking motifs that score very highly for phosphorylation consensus conservation are noted. ConDens scores can found in S6 Data.

We assigned two ConDens scores to each protein encoded by the *S*. *cerevisiae* proteome: one for Cbk1 core docking peptides and another for Cbk1 phosphorylation consensus sequences. Comparing these scores identifies six proteins with clear conservation of both short linear motifs (Fig 7). There is experimental evidence for in vivo phosphorylation of Cbk1 consensus sites in all six of these proteins, and for physical interaction with Cbk1 for most of them [22,23,49,50]. This analysis strongly suggests that, in addition to Ace2 and Ssd1, the proteins Boi1, Fir1, Irc8, and Bop3 are in vivo targets of Cbk1 that engage in docking interactions with the kinase. Tao3, a RAM network component that physically interacts with Cbk1, contains a core docking motif with a high ConDens score, as well as Cbk1 phosphorylation consensus sequences whose ConDens scores are relatively low because they occur in a region of high background conservation. Intriguingly, the proteins Dsf2, Mpt5, and Sec3 lack a Cbk1 docking motif but have Cbk1 phosphorylation consensus sequences that are clearly conserved; there is evidence for in vivo phosphorylation of these sites in Mpt5 and Sec3 [49,50].

We found that proteins with a match to the [YF]xFP Cbk1 core docking motif are significantly more likely to contain matches to the Cbk1 phosphorylation consensus motif (S9 Fig) (Fisher’s exact test, *p* = 0.016). In proteins in which the core docking motif is conserved, the probability is dramatically higher that Cbk1 phosphorylation consensus sequences are present, and these consensus sites are themselves far more likely to be conserved (Fisher’s exact test, *p* < 10^−6^ and *p* < 10^−10^, respectively). Furthermore, consensus site matches in proteins with conserved Cbk1 core docking motifs are significantly more likely to be phosphorylated in vivo (PhosphoGrid annotation [50]; *p* = 0.01) (S7 Fig). Thus, Cbk1’s core docking motif appears to be a useful predictor of functionally important Cbk1 phosphorylation consensus motifs and previously unappreciated regulatory targets of the budding yeast RAM network.

## Discussion

### Cbk1–Mob2, a Structural Template for an NDR/LATS Kinase–Coactivator Complex, Provides Insight into Activation Mechanisms

Structural and biochemical analysis of the yeast Cbk1–Mob2 complex presents a picture of a dynamic and allosterically regulated molecular switch, in which a distinctive activation loop and N-terminal kinase domain extension superimpose regulatory mechanisms that are unique to NDR/LATS kinases on a highly conserved AGC kinase catalytic core. For example, regions of Cbk1’s activation loop conserved among the NDR/LATS family may directly block substrate binding when the kinase is inactive, while autophosphorylation of the flexible region promotes a restructuring that brings the kinase into an “open” state that can bind substrates. Additionally, the NDR/LATS N-terminal extension adopts a novel conformation upon association with Mob coactivator proteins, forming a dramatic cleft where the two proteins come together.

Our analysis indicates that Mob coactivator association creates a mechanism that discriminates between the phosphorylated and unphosphorylated states of the NDR/LATS HM. In all of our crystal structures, Cbk1’s HM region is bound within the cleft formed by Mob association with Cbk1’s N-terminal extension. This organization differs from that of PKB/Akt, in which HM site phosphorylation promotes its association with the PIF pocket on the kinase N-lobe, shifting the αC helix to a conformation that organizes the catalytic site in an optimally active state [39,51]. Our MD simulations suggest that phosphorylation of the NDR/LATS HM site leads to compression of the N-linker region and allows bidirectional interactions between the αMob helix, the HM, and a site analogous to the PIF pocket. This interaction depends on the presence of the Mob cofactor.

**Fig 8:**
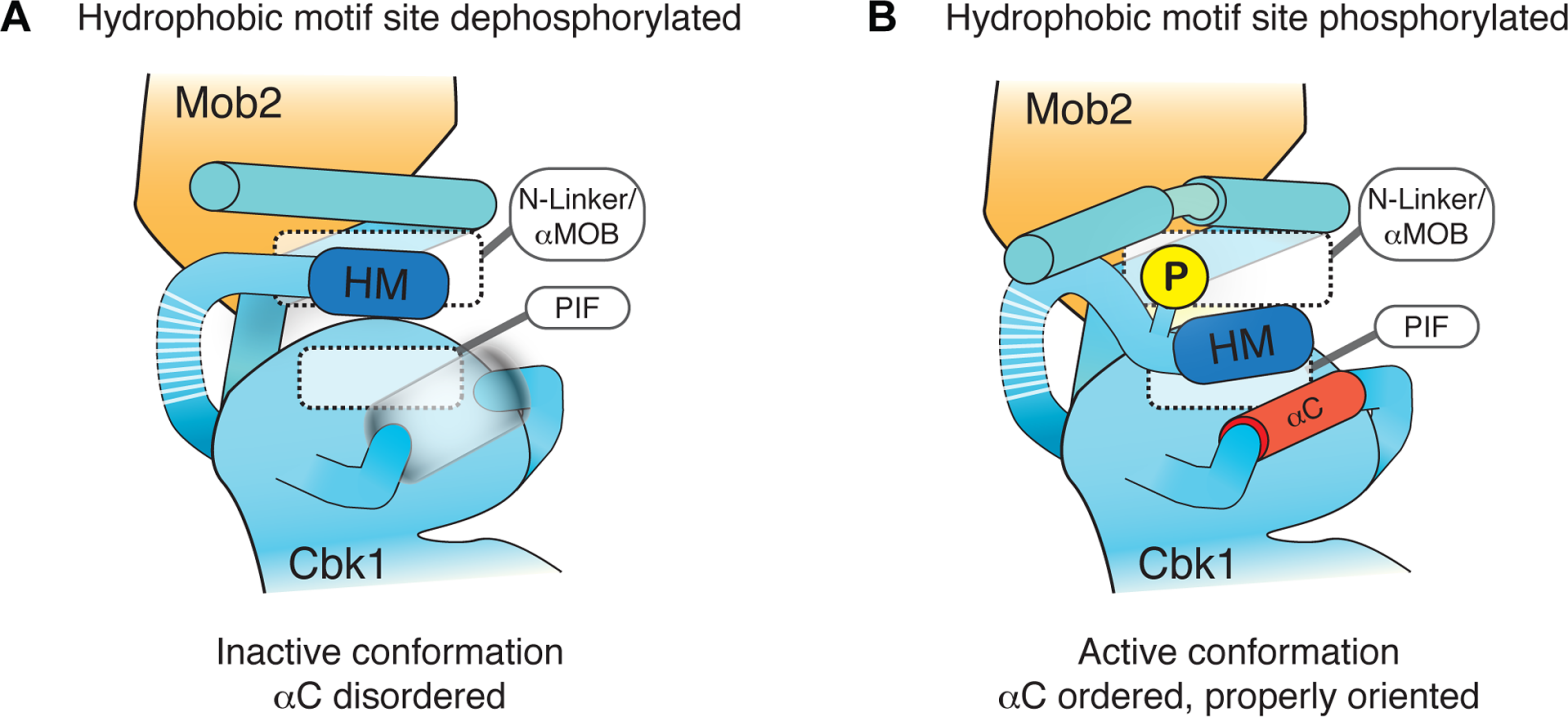
Model for role of Mob binding and HM phosphorylation in NDR/LATS kinase activation. Mob supports the NDR/LATS kinase N-terminal extension in a conformation that creates a binding slot for both HM-T and HM-P. (A) HM-T remains associated with the N-linker region in a manner that does not promote an ordered conformation of the αC helix. (B) HM-P promotes reorganization of αMob, compressing the HM binding slot and holding aromatic side chains of the HM in a configuration that favors ordering of αC.

Based on these data, we propose that the unphosphorylated NDR/LATS HM (HM-T) binds the kinase N-linker region, but cannot shift to a conformation that orders αC. The phosphorylated form of the HM site (HM-P) can also bind in this manner, but causes significant reorganization of the cleft formed by Mob2 association, leading to active kinase (Fig 8). Intriguingly, replacing human NDR’s HM region with the corresponding segment of PKB/Akt yields a hyperactive kinase that does not appear to require its Mob coactivator [52]. In the context of our findings, this suggests that the NDR/LATS kinases have evolved an activating motif that requires Mob interaction. Ordering of αC in the activated state of Cbk1 is a key element of this activation model. Although our inactive Cbk1–Mob2 crystallographic complexes do not suggest how this mechanistically may happen, it is likely that the HM motif will dock onto the αC helix, based on other activated AGC kinase structures. Cbk1 activation may be reminiscent of the structurally characterized activation model of RSK, where activating phosphorylation grossly remodels the αC of its AGC kinase domain [53].

Hippo pathways, characterized in animals and yeast, require large scaffold proteins to spatially coordinate network components and efficiently activate the NDR/LATS kinase [1,6]. The yeast MEN pathway, for example, requires the scaffold Nud1 for Cdc15 to activate Dbf2, whose recruitment is facilitated by a Mob1 phospho-recognition region that Mob2 lacks [12,14]. The scaffold for the RAM network is currently unknown, although the large protein Tao3 that is related to metazoan furry family proteins has genetic interactions with all RAM components and may fulfill that role. The need for scaffolding is bolstered by the fact that the HM site is a poor substrate for the upstream hippo kinase [25]. Our structures show that there is likely an autoinhibitory HM binding site in the N-linker region, which would further hinder HM phosphorylation. We suspect scaffolding may not only function to bring hippo components together, but is likely also necessary to allosterically regulate HM N-linker region dynamics to facilitate efficient hippo phosphorylation and thus NDR/LATS activation.

Overall, the structures of Cbk1–Mob2 complexes suggest that Mob coactivator binding by the NDR/LATS kinases enhances the general HM allosteric mechanism that is a hallmark of AGC kinase regulation, making it more switch-like. This provides an elegant example of a common regulatory mechanism altered and adapted by the presence of extra regulatory factors. This fits well with the physiological roles of NDR/LATS kinases as central regulators of cell-cycle-dependent processes. Therefore, physiologically and mechanistically, the NDR/LATS–Mob coactivator system is reminiscent of the far better characterized cyclin-dependent kinase (CDK)–cyclin system [45,54].

### Cbk1’s Docking Mechanism Suggests a Surface at Which New Substrate Interaction Specificity Evolves

We have shown that a site on the C-terminal lobe of Cbk1’s catalytic domain interacts with a short peptide docking motif in the kinase’s known in vivo substrates, Ace2 and Ssd1. AGC kinase catalytic domains have not thus far been demonstrated to bind short linear docking motifs in substrate proteins in this way. In one case, the AGC kinase PDK1 uses a form of substrate docking to associate with PKB/Akt [29]. This system, however, is an adaptation of an intramolecular regulation mechanism for recruitment of a regulatory target: PDK1 lacks its own cis-regulatory C-terminal HM region, making available a site where the corresponding HM region of PKB/Akt associates. Our findings describe a form of substrate docking that has not been seen previously in an AGC group kinase, and thus raise the possibility that similar mechanisms may be more widely distributed.

Cbk1’s docking peptide binding site is built from conserved structural elements and, in a broad sense, is located similarly to the well-characterized D motif binding region of MAPKs such as ERK and JNK [55,56]. As in the MAPKs, part of Cbk1’s peptide-binding site is formed by a short loop that links the D and E alpha helices of the core catalytic domain. Another crucial part of Cbk1’s docking peptide binding site is contributed by the kinase’s conserved AGC kinase C-terminal extension, in the CLT [28]. Here, aromatic amino acids near a pair of prolines in a conserved PxxP motif play an important role in Cbk1’s docking motif binding surface.

The similarities between the Cbk1 peptide docking site and the D motif binding site of MAPKs, which are members of the relatively distantly related CMGC group, are intriguing. However, D motif peptides that associate with MAPKs are unrelated to the core docking motif that binds Cbk1, and the topography of the peptide–kinase interaction is different (S8A Fig). Furthermore, the amino acids in the D–E loop are divergent between Cbk1 and the MAPKs. Thus, the relationship of the docking motif interaction sites of Cbk1 and the MAPKs indicates that this kinase surface is probably functionally well suited for peptide interaction, with distinct sequence specificity arising in different lineages.

The involvement of Cbk1’s CLT in docking motif binding suggests that other AGC kinases might also use this region to associate with peptide motifs in target proteins. The overall organization of Cbk1’s docking surface is fairly similar to those of PKB/Akt and ROCK [36,51] (S8B Fig). In fungal NDR/LATS kinases, the combination of bulky aromatic side chains important for Cbk1’s docking peptide binding is clearly present. However, while the kinase regions that underlie the Cbk1 docking surface are present in metazoan orthologs, there is considerable evolutionary plasticity within them (S9 Fig). For example, the PxxP motif that occurs in the CLT of LATS and most AGC kinases has been lost in metazoan NDR orthologs. The D–E loop is also evolutionarily divergent. Thus, while the protein surface Cbk1 uses for docking peptide binding is commonly present in a wide range of protein kinases, its ability to specifically bind the [YF]xFP motif probably arose in the evolution of this NDR family kinase in fungi. The variability of amino acids clustered at this kinase surface suggests that it is a place where new peptide interaction specificities can evolve. We therefore propose that other members of the NDR/LATS family and the broader group of AGC kinases adopt analogous docking solutions, albeit with different specificity.

### New Cbk1 Targets Implicate Additional Processes in Cell Separation and Morphogenesis

**Fig 9:**
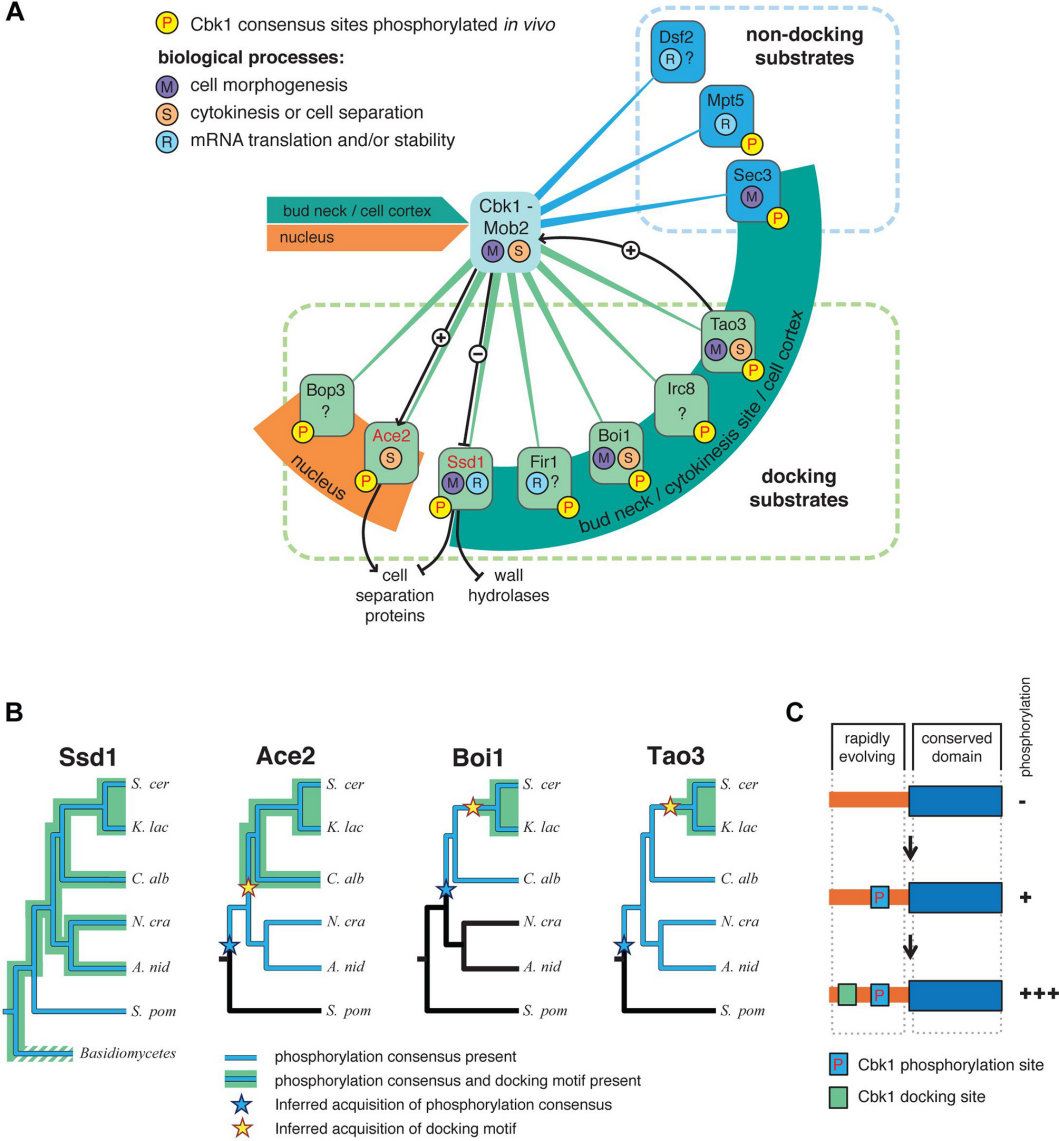
A probable network of proteins controlled by the yeast RAM network, and sequential evolution of docking in Cbk1 substrates. (A) An outline of known and likely Cbk1 substrates in which the core docking motif is conserved (green lines) and likely substrates that lack a docking motif (blue lines). Ace2 and Ssd1 (red type) are known Cbk1 phosphorylation targets, and black lines indicate regulatory interactions inferred from molecular genetic analysis. Orange and green arcs indicate localization of target proteins to either the nucleus or sites of cell growth and cortical remodeling (bud neck/cytokinesis site/cell cortex): Cbk1 displays both of these localization patterns. (B) Distribution of phosphorylation consensus sites and docking motifs in known and likely Cbk1 substrates. Orthologs of Ace2, Boi1, and Tao3 are present in the lineages drawn. Blue indicates lineages in which the proteins have conserved phosphorylation consensus sites, while in those drawn in black, consensus sites are absent. Inferred acquisition of consensus sites is represented by a blue star. Lineages in which the proteins have conserved Cbk1 docking motifs are highlighted green, with inferred acquisition of this motif labeled by a yellow star. (C) Sequential addition model for acquisition of regulatory motifs during signaling system evolution. Substrates first become phosphorylation targets of a given upstream kinase. Docking motifs are subsequently acquired in unstructured, rapidly evolving sequence. The robustness of substrate phosphorylation increases upon docking motif acquisition. *A*. *nid*, *Aspergillus nidulans*; *C*. *alb*, *Candida albicans*; *K*. *lac*, *Kluyveromyces lactis*; *N*. *cra*, *Neurospora crassa*; *S*. *cer*, *Saccharomyces cerevisiae*; *S*. *pom*, *S* *chizosaccharomyces* *pombe*.

Shared conservation of Cbk1’s docking and consensus motifs can be used to predict proteins that are controlled by this NDR/LATS kinase. This analysis identifies seven proteins that form a putative system of docking substrates (Fig 9A, green lines). As noted, there are also proteins with highly conserved Cbk1 consensus sites but no docking motif, and we propose that these are also regulatory targets of the kinase as a group of non-docking substrates (Fig 9A, blue lines). For nearly all of these proteins, there is evidence that Cbk1 consensus sites are phosphorylated in vivo.

This expanded system of proposed RAM-network-regulated proteins forms a remarkable nexus around the processes of cell morphogenesis, cytokinesis, and cell separation. Several are involved in bud growth and the remodeling of the cell cortex, consistent with their localization. Boi1 acts with proteins involved in cell polarity to help maintain cell polarity, and Ssd1 modulates translation of proteins required for both cell separation and normal expansion of the cell wall [22,57,58]. Additionally, Boi1 has been proposed to function in a regulatory system that prevents completion of cytokinesis when chromosome segments remain in the zone of cell division [59]. Sec3, a predicted Cbk1 substrate that lacks a docking motif, localizes to the bud cortex to recruit proteins that promote polarized secretion and thus localized cell growth. Ace2, which is activated by Cbk1, has a well-established role in the separation of mother and daughter cells [18,19]. While Irc8’s function is unknown, it is a putative membrane protein that localizes to the cortical region of growing buds [60].

The Cbk1 substrate system we propose is also connected to processes of gene expression, mRNA stability, and translational control. In addition to Ssd1’s suppression of translation, Mpt5 promotes mRNA decapping and deadenylation, and genetic interaction suggests that Cbk1 inhibits Mpt5 [61,62]. Fir1’s function is unknown, but it may play a role in mRNA polyadenylation; Bop3 is similarly uncharacterized, although its nuclear localization may indicate a role in transcriptional regulation. Intriguingly, there is evidence for functional integration of some of these proteins. Ace2 drives expression of cell separation genes that are translationally controlled by Ssd1, and this may form a Cbk1-controlled feed-forward loop that is modulated by kinase docking interactions [59].

### A Sequential Model for Adaptive Acquisition of Kinase Docking

The amino acids that are important for Cbk1’s docking motif binding are generally conserved among fungal NDR kinases, suggesting that this interaction mechanism emerged early in the evolution of this branch of life and conferred fitness. Consistent with this, Ssd1 orthologs from basidiomycetes to ascomycetes have the core docking motif and Cbk1 phosphorylation consensus motifs in a conserved organization, although in some lineages the docking motif is absent (Fig 9B).

While Cbk1’s docking interaction appears ancient in fungi, the kinase’s probable substrates do not invariably have docking motifs (Figs 7 and 9A). It is therefore plausible that proteins first become kinase substrates and then subsequently evolve docking. In this view, kinase docking motifs arise by random evolution of unconstrained regions in proteins that are already Cbk1 substrates, and are retained if they enhance the regulatory interaction. This is consistent with the observation that short functional motifs evolve rapidly in intrinsically disordered segments of proteins [63]. To test this model we inferred the ancestral configuration of Cbk1 phosphorylation consensus sequences and docking motifs in known and likely Cbk1 substrates, where we could confidently identify orthologs across a wide span of fungal evolution.

For at least three known or likely Cbk1 substrates, consensus phosphorylation sites were clearly present before the core docking motif appeared in unstructured protein regions (Fig 9B). In these cases, there are orthologs in species that diverged from the lineage leading to *S*. *cerevisiae* that have phosphorylation sites, but no docking motifs. In other likely Cbk1 substrates, phosphorylation sites and docking motifs appear simultaneously. We never found cases in which the appearance of docking motifs predates the acquisition of phosphorylation sites. We surmise that a surface capable of binding to a [YF]xFP docking peptide emerged in fungal NDR and provided selection for subsequent acquisition of cognate docking motifs in phosphorylation targets, exemplifying co-evolution of kinases and substrates. This kind of adaptive evolution of kinase docking (Fig 9C) is consistent with an increase in signaling complexity that has been proposed as a molecular mechanism underlying the evolution of animal development [64,65].

## Materials and Methods

### Protein Purification

His6-Cbk1^251–756^ was co-expressed with GST-Mob2 (full length or 45–287). Cbk1 kinase domain alone (352–756 or 352–692) was expressed as GST-Cbk1-His6. All constructs were bacterially expressed and purified on a Ni-NTA resin (Qiagen) followed by glutathione Sepharose beads (GE Biosciences). For crystallization, tags were cleaved with TEV protease followed by cation exchange chromatography on a Resource S column. All Cbk1 preparations were subsequently dialyzed into Cbk1 buffer (20 mM Tris-HCl, 150 mM NaCl, 2 mM DTT [pH 8.0]).

Cbk1 substrates containing the docking motifs or substrate sites were bacterially expressed as MBP or GST fusions. For pulldown assays, MBP-fusion lysates were incubated with amylose resin (New England Biolabs) on ice for 15 min, followed by washing with PBS (137 mM NaCl, 2.7 mM KCl, 4.3 mM Na_2_HPO_4_, 1.4 mM KH_2_PO_4_ [pH 7.3]). GST fusions were purified on glutathione Sepharose beads followed by dialysis into Cbk1 buffer.

### Crystallization and Data Collection

The crystal structure of the Cbk1–Mob2 complex was solved in three different crystal forms using Cbk1 constructs with an intact or with a mutated (T743E) HM motif (S1 Table).

To determine the structure of the Cbk1–Mob2 complexes, 4–6 mg/ml protein samples were supplemented with 2 mM AMPPNP, 2 mM MgCl_2_, and 2 mM TCEP. All samples used for crystallization contained an inactivating mutation (D475A) in Cbk1, while some Cbk1 constructs also contained a phosphorylation-mimicking mutation in the HM (T743E). The rational for the D475A mutation was to eliminate the autophosphorylation activity of Cbk1, while the T743E mutation was introduced to capture the kinase in an activated state. Crystallization conditions were found by using a custom in-house PEG crystallization screen in microbatch under oil (1:1 silicon oil:paraffin oil) set-ups at 23°C. All crystals were treated with 10% glycerol before flash freezing in liquid nitrogen. Data on frozen crystals were collected at 100 K on the PXI and PXIII beam lines of the Swiss Light Source (Villigen, Switzerland) using a beam of 1.00 Å wavelength (S1 Table).

Single tetragonal crystals of Cbk1(D475A/T743E) complexed with Mob2 were grown in 25% PEG 20000 buffered with 0.1 M sodium citrate (pH 5.5); however, these crystals diffracted only to 4.5 Å resolution. Hoping to improve the diffraction limit of this low diffracting complex by addition of a docking peptide, crystallization trials were set up in the presence of the Ssd1 docking peptide (TTEQSDFKFP) in approximately 4-fold excess. Plate-shaped crystals could be grown under such conditions with both the Cbk1(D475A/T743E)–Mob2 and the Cbk1(D475A)–Mob2 complexes. These crystals diffracted to 3.6 and 3.3 Å resolution (S1 Table). Unfortunately, we could not locate the peptide in the electron density. It is likely that peptides could not bind in the crystal to Cbk1, as the docking surface was occluded in both crystal forms: Phe447 made prominent hydrophobic crystal contacts to symmetry-related molecules. All crystals contained AMPPNP.

### Data Processing and Refinement

Data was processed with XDS [66], and the structure was solved by molecular replacement with PHASER [67]. The 3.6 Å resolution Cbk1(D475A/T743E) (crystal form B) dataset processed in C222 was used to search with the ROCKI kinase domain, which is highly similar to that of Cbk1 [36] (PDB ID: 2ETR). This gave a clear solution for the Cbk1 kinase domain (TFZ = 14.5, LLG = 184), and the Mob2 subunit was located using the highly similar Mob1 protein [33] (PDB ID: 2HJN) as the search model (TFZ = 6.9, LLG = 175). Later refinement, indicated that the correct space group for this crystal was C2; therefore, its asymmetric unit contains two Cbk1–Mob2 complexes. The MR search with the assembled Cbk1–Mob2 complex model then identified a single complex per asymmetric unit in the 4.5 Å resolution Cbk1(D475A/T743E)–Mob2 crystal (crystal form A). Similarly, only one complex was located in the crystal of the Cbk1(D475A)–Mob2. In contrast, two complexes were found per asymmetric unit in the higher resolution crystals of Cbk1(D475A/T743E)–Mob2. Although all four structural models for the Cbk1–Mob2 complex are subject to different crystal packing interactions, they all display the same Cbk1 and Mob2 domain orientation (S2A Fig). Structure refinement was carried out in PHENIX [68] using secondary structure restraints and translation-libration-screw (TLS) parameterization. Modeling was done manually in Coot [69]. All models have less than 1.5% of residues falling into disallowed regions on the Ramachandran plot. Parts of the electron density maps around the HM motif and the αINH regions are shown in S2B, S2C, S2E, and S2F Fig.

For the Cbk1(D475A)–Mob2 model, which was refined to 3.3 Å resolution, 218 of the 745 residues, corresponding to close to one-third of the used protein constructs, are missing in the final model. For example, a long N-terminal Cbk1 region (251–293) and the N-terminal Mob2 region (45–110) could not be located in the electron density map. Apart from these apparently flexible N-terminal protein construct ends, there was no density for the region corresponding to the αC (392–406), the middle part of the Cbk1 activation loop (508–553), the linker region connecting the Cbk1 kinase core with the hydrophobic C-terminal region (714–739), and an internal Mob2 region (149–160) (see Figs 2A and S1). Unfortunately, all attempts to express shortened constructs failed because the yield of soluble protein was insufficient for structural studies.

### Molecular Dynamics Simulations

First, we constructed a hybrid model that incorporated regions from Cbk1–Mob2 and PKB/Akt crystal structures. Cbk1’s disordered region containing αC was completed with the ordered region from the PKB/Akt’s crystal structure (PDB ID: 1O6K) using homology modeling followed by energy minimization to remove minor clashes. Furthermore, Cbk1’s short activation loop that contains the S570 autophosphorylation site was remodeled to match the position of the phosphorylated activation loop of PKB. The long activation loop (499–570) and the kinase core–HM connector region (714–739) were built in manually in an extended conformation. We subjected this complete model to MD simulations. Initial analyses suggested that the artificially built-in flexible loop regions (499–570 and 714–739) showed too much structural fluctuation, which precluded free MD simulations. Next, we excluded the activation loop (499–570) and performed a 100-ns MD run on the model where only the HM connector loop region (714–739) was allowed to move freely to pre-equilibrate this loop region. All MD simulations started out from this pre-equilibrated model.

Heavy atom position restraints were applied on the C-terminal kinase core region (569–670); otherwise, the structure was allowed to move freely during 125-ns-long MD runs. Protein structures were blocked with N-methyl and acetyl groups at the artificial chain break between Y569 and P670, and the nucleotide was modeled as ATP. In all calculations the GROMACS ver. 4.5.5 program package was used [70], and the Amber-03 force field [71] was applied, along with neutralizing Na+ counter-ions and numerous TIP3P [72] explicit water molecules filling a 5 Å spacing between the protein parts and the edges of the cubic simulation box. Long-range electrostatics was calculated by the PME method and a van der Waals cutoff of 9 Å was used. Prior to the productive MD runs, the systems were energy-minimized using steepest descent and conjugate gradient molecular mechanics energy minimizations with a step size of 0.1 Å, and a tolerance of 10 kJ mol^−1^ nm^−1^. The energy-minimized systems were subjected to MD calculations. A time step of 2 fs, constant temperature of 300 K, and LINCS bond constraints [73] were applied. Neighbor lists were used and updated every 10 fs. Temperature coupling was carried out using the v-rescale scheme [74]. The resulting trajectory files were analyzed with programs of the GROMACS package.

### In Vivo Analysis of RAM Network Output

For cell separation assays, *ckb1*Δ, *ace2*Δ*CBK1*, *or ace2*Δ*cbk1-as2* cells were transformed with empty, WT, or mutant vector under control of the ADH1 promoter. Cells were grown to log-phase, briefly sonicated, and mounted on slides to count cell group size. Histograms represent mean ± standard deviation of three independent cultures, with >100 groups counted for each. For quantification of transcriptional output, RNA was isolated from log-phase cultures and analyzed by real-time PCR. Bar graphs represent mean ± standard deviation of three independent cultures. For growth assays, WT *CBK1* or *cbk1-as2* strains were transformed with vectors containing Ssd1 (WT and mutants) expressed from a galactose-inducible promoter. Stationary phase cells grown in glucose were diluted to OD_600_ = 0.2. Five-fold serial dilutions (5^−2^, 5^−3^, 5^−4^, 5^−5^) were plated in the presence of glucose or galactose, then incubated for 3 d at 24°C.

### In Vitro Pulldown Assays

MBP fusions were incubated on bead with 1 μM purified Cbk1 on ice for 15 min. Samples were washed 3× with PBS (transferred to fresh tube on last wash), resuspended in SDS-PAGE buffer, and separated by SDS-PAGE. Samples were directly visualized with GelCode Blue (Pierce) or transferred to PVDF membrane for immunoblotting. Cbk1 was detected using His or pS570 primary antibodies. For experiments detected using α-pS570, antibody signal with pure protein was analyzed to control for lot-dependent variations in expression as well as variations between Cbk1 mutants (no variations detected).

### In Vitro Kinase Assays

Kinase assays resolved by SDS-PAGE were conducted with GST-Ace2 or GST-Ssd1 fusions. Reactions contained 1–5 μM substrate, 100–250 nM Cbk1, 5 mM MnCl_2_, 2 mM DTT, 20 μM ATP, 10 μCi γ-^32^P-ATP in Cbk1 buffer. Samples were removed at indicated time points and quenched with SDS-PAGE buffer, followed by SDS-PAGE separation. ^32^P was visualized using a Storm phosphorimager (GE Biosciences) and quantified using ImageQuant software.

For peptide kinase assays, reactions were performed in the presence of 50 nM Cbk1^251–756^–Mob2, 40 μM ATP, 2 mM DTT, 5 mM MnCl_2_, 2 μCi γ-^32^P-ATP, and 100 μM Docktide (CDFKFPPPPNAHGGHRRATSN) or AKAPtide (CDAKAPPPPNAHGGHRRATSN). Reactions were initiated by the addition of ATP. Samples were quenched after 1 h by the addition of a 5-μl sample to P81 paper (Whatman) and subsequent submersion in 75 mM phosphoric acid to remove background γ-^32^P-ATP. After drying, signal was determined by Čerenkov counting in an LS 6500 scintillation counter (Beckman). Counts per minute was efficiency corrected and converted to femtomoles ^32^P followed by conversion to total ATP (labeled and unlabeled). Significance was determined by an unpaired two-tailed Student’s *t*-test.

### Fluorescence-Polarization-Based Protein–Peptide Binding Affinity Measurements

For FP-based binding affinity measurements, different docking peptides (pepAce2: GSGSIIVTTNSANGGYQFP; pepSSD1: TTEQSDFKFP; pepHM: IGYTYSRFDY; pepHM-P: IGYpTYSRFDY) were N-terminally (pepAce2 and pepSSD1) or C-terminally (pepHM and pepHM-P) labeled with carboxyfluorescein (CF) fluorescent dyes.

Change in the FP signal in direct binding affinity measurements was monitored as a function of increasing concentration of protein with a Synergy H4 (BioTek Instruments) plate reader in 384-well plates. The labeled peptides were at 20 nM in 20 mM Tris (pH 8.0), 100 mM NaCl, 0.05% Brij35P, 2 mM DTT. The resulting binding isotherms were fit to a quadratic binding equation. The affinity of the unlabeled peptides was measured in steady-state competition experiments: 20 nM labeled reporter was mixed with protein samples in a concentration to achieve ~60%–80% complex formation. Subsequently, increasing amounts of unlabeled peptide were added, and the FP signal was measured as described earlier for direct titration experiments. The K_d_ for each unlabeled peptide interaction was determined by fitting the data to a competition binding equation. Titration experiments were carried out in triplicate, and the average FP signal was used for fitting the data with OriginPro 7.

### Identification of the Cbk1 Docking Surface

The structure of acetyl-DFKFP-NH-methyl ligand was built and optimized using the Tinker program package [75] and the Amber force field [76] with an automatic selection of minimum search algorithms and a gradient norm of 0.001. The target protein was the Cbk1 kinase domain from the 3.3 Å resolution Cbk1–Mob2 crystal structure where missing side chains were inserted automatically by rotamer preferences in Coot and the missing αC helix was homology modeled based on the corresponding region from PKB. The completed structure was energy-minimized using the GROMACS software package [77] using Amber force field, TIP3P explicit water surrounding, steepest descent, and conjugate gradient optimizations with tolerances of 1,000 and 100 kJ mol^−1^ nm^−1^, respectively. Notably, in the docking calculations the highly unstructured loop between residues Y509 and S552 was not used to avoid docking artifacts, and the terminating residues were mutated into chain-blocking—NH-methyl and acetyl—ends, respectively. We prepared both target and ligand structures for docking with the aid of AutoDockTools [46]. We performed blind docking using AutoDock 4 [78] at 0.375 Å grid spacing in a docking box of 93.8 × 93.8 × 93.8 Å^3^ (250 grid points in each direction) covering the entire target. The number of energy evaluations was increased to 50 million, and the population size of the genetic algorithm was set to 250 to handle the completely flexible ligand with 20 active torsions (only omega torsions of backbone amide groups were kept restrained in trans conformation). More than 200 docking runs were performed and evaluated, and other parameters not described above were set as in previous studies [78–80]. We performed focused docking similarly with an acetyl-FKFP-NH-methyl tetrapeptide using a smaller docking box (63.8 × 75.0 × 63.8 Å^3^) centered at a point (−61.65, 41.95, 5.8) above the docking interface found by blind docking. Unbiased and focused docking resulted in the same binding conformation for the FKFP core motif (Fig 4A).

Binding experiments with constructs containing mutated Ssd1 docking motifs showed that for the FxFP core motif, the first-position tyrosine and phenylalanine are equivalent, but only phenylalanine is tolerated at the third position (Fig 4D and 4E). There is also a positively charged amino acid (lysine or arginine) requirement for the second position. The final model is in excellent agreement with experimental observations on the importance of Ssd1 and Cbk1 docking surface residues for Cbk1–Ssd1 binding (see Figs 4 and S5).

### Analysis of Docking Motif and Phosphorylation Consensus Site Conservation

To identify proteins with conserved phosphorylation consensus sites or conserved docking motifs we used ConDens [48] with alignments of related ascomycetes taken from the Yeast Gene Order Browser [81]. Briefly, ConDens compares the density of matches to the specified short peptide sequence in each species to the density of matches in a “reference” species (in our case *S*. *cerevisiae*) and evaluates the probability that each species retained as many matches (or more) to that sequence in the absence of selection. Each species and each site in the protein are then combined into a single score (shown in Fig 6) for each protein in *S*. *cerevisiae*.

### Accession Numbers

Coordinates and structure factors for the reported crystal structures have been deposited to the PDB under accession codes 4LQP, 4LQQ, and 4LQS.

## Supporting Information

S1 Data Measurements of the distances between the centers of mass of indicated Cbk1 and Mob2 regions over the course of the molecular dynamics simulations described in Figs 3 and S4. This file also contains data quantifying the effectiveness of cell separation in different genetic backgrounds. In these experiments we counted the number of cells present in clumps of specific sizes in random microscope fields, and then binned these groups into clump size ranges: 1–4 cells, 5–10 cells, 11–20 cells, and ≥21 cells per clump. We quantified this for the indicated allele combinations, scoring three independent isolates of each allele combination. The number of cell bodies present in clumps of different sizes is a function of the efficiency of cell separation.
(XLSX)

S2 Data Fluorescence polarization assay data that are represented in graphical form in Figs S3 and S5. (XLSX)

S3 Data Enzymological characterization of the effects of Cbk1 docking. Assay data from experiments that used γ-^32^P-ATP in protein kinase reactions with synthetic substrate peptides from Ssd1 that either contained both the core Cbk1 docking motif and a Cbk1 phosphorylation consensus motif (Docktide) or contained a core Cbk1 docking motif mutated to abrogate binding to the kinase and a Cbk1 consensus motif (AKAPtide). These kinase assays were performed either with Cbk1(T743E) or with Cbk1(Y687A/T743E). The latter construct carries a mutation that eliminates docking motif association with the kinase.
(XLSX)

S4 Data Data quantifying the effectiveness of cell separation in different genetic backgrounds. In these experiments we counted the number of cells present in clumps of specific sizes in random microscope fields, and then binned these groups into clump size ranges: 1–2 cells, 3–4 cells, 5–10 cells, 11–20 cells, and ≥21 cells per clump. We quantified this for the indicated allele combinations, scoring three independent isolates of each allele combination. The number of cell bodies present in clumps of different sizes is a function of the efficiency of cell separation.
(XLSX)

S5 Data Real-time PCR data for experiments measuring mRNA levels of the Ace2 target genes *CTS1*, *DSE1*, and *SCW11*, as well as *ACT1* mRNA in strains carrying different alleles of *CBK1* and *ACE2* (WT, as well as mutant alleles affecting different protein functions). (XLSX)

S6 Data Scores computed using the ConDens algorithm [48], which evaluates conservation of sequence motifs in a manner not dependent on precise alignment of orthologous sequences. This file lists proteins in which Cbk1 phosphorylation consensus and core docking motif sequences are significantly conserved, as well as proteins in which one but not the other of these two sequence motifs is significantly conserved.
(XLSX)

S1 Fig Multiple sequence alignment of NDR kinases. Sequence comparison of Cbk1 from *Saccharomyces cerevisae* (CBK1_YEAST) with other NDR kinases from different organisms (*SCHPO: Schizosaccharomyces pombe; TRIAD: Trichoplax adherens; CAEEL: Caenorhabditis elegans; DROME: Drosphila melanogaster; DANRE: Danio rerio*). The consensus sequence is presented as a sequence logo. Residues and motifs important for Cbk1 activity or for its regulation are boxed: Asp475 is the catalytic aspartate, Ser570 is an autophosphorylation site, while Thr743 is phosphorylated by an upstream kinase. Important regions are underlined. Highlighting is based on sequence identity compared to Cbk1. Secondary structure elements from the crystallographic models are shown above the sequences. Dashed lines indicate regions that could not be built into the crystallographic model of the Cbk1–Mob2 complex.
(PDF)

S2 Fig Additional information on the crystal structures of Cbk1–Mob2 complexes. (A) Superimposition of the crystallographic models for Cbk1–Mob2 complexes. All four complexes (from three different crystal forms) display the same Cbk1 and Mob2 domain arrangements. (B) The Cbk1 kinase domain (shown in blue) is similar to the AGC kinase domains from related kinases such as PKA (PDB ID: 1JLU; yellow) and ROCK1 (PDB ID: 2ETR; green) [36,37]. In contrast, the activation loop and N-terminal kinase domain extensions adopt markedly different structures. (AGC kinase domains are shown in thin ribbon, while activation loops and N-terminal kinase domain extensions are shown in thicker tube representation.) (C) Stereo view of the final 2Fo-Fc electron density map around the activation loop (cyan) at 3.3 Å resolution. (D) In the two different crystal forms of Cbk1(T743E), the αINH is rotated with an angle of ~30° due to different crystal packing. For clarity, Cbk1 from crystal form A is colored purple and from crystal form B is colored in blue. (E) Simulated annealing 2Fo-Fc omit map contoured at 1σ and calculated around the HM motif region for the 4.5 Å resolution Cbk1–Mob2 structure. (F) Omit map (generated with the same protocol) for the 3.3 Å resolution Cbk1–Mob2 crystal structure.
(PDF)

S3 Fig Characterization of the Cbk1 hydrophobic motif and its binding to the Cbk1–Mob2 complex. (A) Summary of the FP binding experiments with Cbk1-HM-containing peptides. Although the Cbk1–Mob2 and the Cbk1(T743E)–Mob2 complexes did not show major changes in the position of their Cbk1 HM motifs, we compared the binding affinity of peptides containing unphosphorylated and phosphorylated HM motifs to a Cbk1^∆730–756^ variant complexed with Mob2 (which lacks the HM region) in trans. These in vitro binding affinity measurements indicated that the phosphorylated HM peptide bound more than 5-fold stronger in trans into the open Cbk1 HM-binding slot. Binding of a carboxyfluorescein-labeled Cbk1-HM-containing peptide was monitored and compared to the binding of the phosphorylated HM peptide. In direct titration binding experiments, the binding of the labeled peptide was monitored and binding isotherms were fit to a classical binding equation. Competitive titration experiments (starting from 60%–80% complex formation between the protein and the labeled peptide) monitored the binding of unlabeled peptide as it competed with the labeled peptide for the same binding site. Competitive titrations can indirectly report on the binding affinity of unlabeled peptides if data are fit to a competition binding equation. ND, not determined. (Note that these experiments monitored the binding of peptides in trans, and they do not report on the binding of the HM motif as part of the full-length Cbk1.) (B) Phosphorylated HM motif binds to Cbk1^Δ730–756^–Mob2 with increased affinity. (C) Competitive titration curves for monitoring HM and HM-P peptide binding to the Cbk1^Δ730–756^–Mob2 construct. The markedly reduced binding affinity for the unlabeled versus the labeled HM-P peptide is likely due to the presence of the fluorophore, which seems to artificially increase binding. (D) Competitive titration binding curve for monitoring labeled HM-P and Ssd1 docking peptide binding. This panel shows that the docking peptide could not compete with the HM peptide, indicating that the Ssd1 docking peptide does not bind to the groove around the Cbk1 N-linker region. Each measurement is representative of at least two sets of independent experiments where K_d_ values were calculated from triplicate data points. Triplicates were independently prepared samples that were assayed at the same time. Error bars on the measurement points indicate standard deviations from the mean. Errors in the calculated K_d_ values indicate uncertainty of the fit to a direct or competition binding equation. FP data can be found in S2 Data.
(PDF)

S4 Fig Molecular dynamics simulations of Cbk1–Mob2 complexes. (A) Cbk1–Mob2 interface analysis. Upper panel shows the angle distribution between αMOB (Cbk1) and H2 (Mob2) as defined on the left panel. The small differences between the HM-P complex and the HM complex are not statistically significant. Lower panel displays interface area between Cbk1 and Mob2. These analyses show that there is no major global change at the Cbk1–Mob2 interface during MD, confirming that the changes shown in Fig 3 occur because HM-mediated local interactions change within the binding slot. (B) Three-dimensional N-linker–HM, αC–HM and αMOB–HM distance scatter plots on Cbk1 with unphosphorylated and phosphorylated HM. MD simulations were identical to those on Cbk1–Mob2 complexes, but the Mob2 protein chain was removed from the starting MD model. (C) Three-dimensional N-linker–HM, αC–HM, and αMOB–HM distance scatter plots on Cbk1 with HM-E. MD simulations were identical to those on Cbk1–Mob2 complexes, but Cbk1 T743 was mutated to glutamic acid. MD data can be found in S1 Data.
(PDF)

S5 Fig Identification and analysis of docking motifs in Ace2 and Ssd1. (A) Pulldown of Cbk1–Mob2 by Ace2 truncation fragments (left) and Ace2 fragments centered on the docking motif (right). Smaller fragments than Ace2^272–286^ abrogate Cbk1 interaction. (B) Alanine scan of Ace2^270–290^ pulldown with the Cbk1^352–692^ kinase domain alone highlights the importance of N-terminal hydrophobic residues in addition to the C-terminal core motif. (C) Flexibility analysis of Ace2^270–290^ by glycine insertion/deletion and pulldown of Cbk1–Mob2. The bipartite motif can be extended, but deletion abrogates Cbk1 interaction. WT Ace2 contains two glycine residues. (D) Cbk1^352–756^ kinase domain in vitro kinase assay with Ace2^102–306^. The presence of the docking motif enhances phosphorylation 100-fold as well as enhances Cbk1 autophosphorylation. (E) Pulldown of Cbk1^352–756^ by Ssd1 truncation fragments containing the N-terminal (1–6) or C-terminal (7–11) docking motif. (F) Pulldown of Cbk1^352–756^ by the Ace2 docking motif with stepwise conversion to the Ssd1 docking motif. Conversion of the core motif (YQFP → FKFP) could not rescue mutation of N-terminal residues, highlighting the importance of surrounding sequence to the core motif. (G) Competition of Ace2 (left) and Ssd1 (right) docking motifs with unbound Ace2^270–290^. Competition was analyzed by Cbk1^352–756^ pulldown. (H) FP affinity measurements of peptides containing Ssd1 (top) and Ace2 (bottom) docking motifs with Cbk1–Mob2. (I) FP of Ssd1 with the Cbk1 kinase domain (top) or with the HM deleted (bottom). Neither Cbk1 truncation exhibited defects in docking motif interactions. FP data can be found in S2 Data.
(PDF)

S6 Fig Cbk1–Ace2 docking confers robustness to regulation of Ace2 target gene transcription. (A) *CBK1* WT cells carrying *ace2* ^dock*^ have no significant reduction in transcript levels of the *DSE1* gene, while *cbk1-as2* cells carrying *ace2* ^dock*^ exhibit dramatically reduced *DSE1* transcription. (B) *CBK1* WT cells carrying *ace2* ^dock*^ exhibit modest reduction in transcript levels of the *SCW11* gene, while *cbk1-as2* cells carrying *ace2* ^dock*^ exhibit strongly reduced *SCW11* transcription. Transcription data can be found in S5 Data.
(PDF)

S7 Fig Co-occurrence of docking motifs and Cbk1 phosphorylation consensus sites is extremely significant, and docking sites predict consensus site conservation and phosphorylation. (A) In proteins that contain matches to the Cbk1 docking motif [YF]xFP, there is a statistically significant enrichment of NDR/LATS phosphorylation consensus sites Hx[RK]xx[ST] compared to proteins that do not contain docking motifs (0.45 versus 0.36 matches per 1,000 amino acids; Fisher’s exact test, *p* = 0.016). Similar results are obtained for phosphorylation consensus sites for which there is mass spectrometric evidence of phosphorylation as annotated in 2013 by PhosphoGrid [50] (0.040 versus 0.017 phosphorylated matches per 1,000 amino acids; Fisher’s exact test, *p* = 0.013). If we consider the 50 most conserved phosphorylation consensus sites as identified by ConDens [48], the enrichment is much stronger (0.067 versus 0.011 conserved matches per 1,000 amino acids; Fisher’s exact test, *p* = 3.629 × 10^−8^). Since many matches to the docking motif ([YF]xFP) might appear in proteins by chance, we also considered proteins that contain conserved docking motifs. In these proteins the enrichments are much stronger. For example, phosphorylation consensus matches are now >8× more like to appear (2.95 versus 0.36 matches per 1,000 amino acids; Fisher’s exact test, *p* < 10 × 10^−10^). Similarly, phosphorylated consensus matches and the 50 most conserved phosphorylation consensus matches are >30× and >100× more likely to appear (Fisher’s exact test, *p* < 10 × 10^−6^ and *p* < 10 × 10^−10^, respectively). These tests show that a conserved docking site makes consensus sites (and conserved consensus sites) more likely to appear, and that the phospho-acceptor residues within these consensus sites are significantly more likely to be among the set of phosphorylated positions annotated in the PhosphoGrid database. (B) In the 11 proteins with conserved [YF]xFP docking motifs, 14/27 (51.8%) of the Cbk1 phosphorylation consensus matches are in the 50 most conserved, which is much more than 50/887 (5.6%) in the proteome (Fisher’s exact test, *p* < 10 × 10^−10^) Similarly, consensus matches are more likely to be phosphorylated if they are found in proteins with conserved docking motifs (18% versus 5%, *p* = 0.01) These tests show that a given Cbk1 phosphorylation consensus match is much more likely to be conserved and phosphorylated in vivo if it is found in a protein that contains a conserved docking motif. Therefore, conserved docking motifs point to the functional Cbk1 consensus sites. ConDens data can be found in S6 Data.
(PDF)

S8 Fig Comparison of analysis of Cbk1’s docking motif binding site with corresponding regions from MAPKs and other AGC kinases. (A) A comparison of Cbk1 bound to the FKFP core docking motif and ERK2 bound to the MNK1 docking motif (2Y9IQ) [55]. The loop between kinase domain D and E helices is noted for both Cbk1 and ERK2, and Cbk1’s CLT region is also highlighted. An overlay of the structures shows different peptide binding modes on roughly corresponding surface regions of both kinases. (B) Overlay of the Cbk1 docking region with the corresponding surface of PKA (PDB ID: 1JLU; yellow) and ROCK1 (PDB ID: 2ETR; green) [36,37]. Side chains of amino acids differ, but are placed in a roughly similar geometry.
(PDF)

S9 Fig Evolutionary plasticity of the site at which Cbk1 binds to the core docking peptide. A gene tree of AGC kinases related to the NDR family, with the closest Cbk1 orthologs in *S*. *cerevisiae* (Sc), *Sc*. *pombe* (Sp), *D*. *melanogaster* (Dm), and *Homo sapiens* (Hs) bracketed in red. The loop between the D and E helices in the kinase C-lobe and the PxxP motif in the CLT region are enclosed in orange boxes. Bulky hydrophobic amino acids involved in docking motif binding in Cbk1 are boxed in pink. Tree is not drawn to scale. PKA and MAST are used as outgroups to the NDR family.
(PDF)

S1 Table Relevant crystallographic parameters for the three different crystal forms of the Cbk1–Mob2 complex we solved. (DOCX)

## Abbreviations:

CLT: C-lobe tether
FP: fluorescence polarization
HM: hydrophobic motif
MD: molecular dynamics
MEN: mitotic exit network
RAM: regulation of Ace2 and morphogenesis
NCS: noncrystallographic symmetry
PDB: Protein Data Bank
WT: wild-type

## References

[1] Avruch J, Zhou D, Fitamant J, Bardeesy N, Mou F, et al (2012) Protein kinases of the Hippo pathway: regulation and substrates. Semin Cell Dev Biol 23: 770–784. 10.1016/j.semcdb.2012.07.002 22898666 PMC3489012

[2] Hergovich A, Hemmings BA (2012) Hippo signalling in the G2/M cell cycle phase: lessons learned from the yeast MEN and SIN pathways. Semin Cell Dev Biol 23: 794–802. 10.1016/j.semcdb.2012.04.001 22525225 PMC3459816

[3] Hergovich A, Stegert MR, Schmitz D, Hemmings BA (2006) NDR kinases regulate essential cell processes from yeast to humans. Nat Rev Mol Cell Biol 7: 253–264. 16607288 10.1038/nrm1891

[4] Pan D (2010) The hippo signaling pathway in development and cancer. Dev Cell 19: 491–505. 10.1016/j.devcel.2010.09.011 20951342 PMC3124840

[5] Zhao B, Tumaneng K, Guan KL (2011) The Hippo pathway in organ size control, tissue regeneration and stem cell self-renewal. Nat Cell Biol 13: 877–883. 10.1038/ncb2303 21808241 PMC3987945

[6] Enderle L, McNeill H (2013) Hippo gains weight: added insights and complexity to pathway control. Sci Signal 6: re7. 24106343 10.1126/scisignal.2004208

[7] Cornils H, Kohler RS, Hergovich A, Hemmings BA (2011) Downstream of human NDR kinases: impacting on c-myc and p21 protein stability to control cell cycle progression. Cell Cycle 10: 1897–1904. 21593588 10.4161/cc.10.12.15826

[8] Emoto K, He Y, Ye B, Grueber WB, Adler PN, et al (2004) Control of dendritic branching and tiling by the Tricornered-kinase/Furry signaling pathway in Drosophila sensory neurons. Cell 119: 245–256. 15479641 10.1016/j.cell.2004.09.036

[9] Geng W, He B, Wang M, Adler PN (2000) The tricornered gene, which is required for the integrity of epidermal cell extensions, encodes the Drosophila nuclear DBF2-related kinase. Genetics 156: 1817–1828. 11102376 10.1093/genetics/156.4.1817 PMC1461384

[10] Cornils H, Kohler RS, Hergovich A, Hemmings BA (2011) Human NDR kinases control G(1)/S cell cycle transition by directly regulating p21 stability. Mol Cell Biol 31: 1382–1395. 10.1128/MCB.01216-10 21262772 PMC3135299

[11] Bardin AJ, Amon A (2001) Men and sin: what’s the difference? Nat Rev Mol Cell Biol 2: 815–826. 11715048 10.1038/35099020

[12] Rock JM, Amon A (2011) Cdc15 integrates Tem1 GTPase-mediated spatial signals with Polo kinase-mediated temporal cues to activate mitotic exit. Genes Dev 25: 1943–1954. 10.1101/gad.17257711 21937712 PMC3185966

[13] Wurzenberger C, Gerlich DW (2011) Phosphatases: providing safe passage through mitotic exit. Nat Rev Mol Cell Biol 12: 469–482. 10.1038/nrm3149 21750572

[14] Rock JM, Lim D, Stach L, Ogrodowicz RW, Keck JM, et al (2013) Activation of the yeast Hippo pathway by phosphorylation-dependent assembly of signaling complexes. Science 340: 871–875. 10.1126/science.1235822 23579499 PMC3884217

[15] Weiss EL (2012) Mitotic exit and separation of mother and daughter cells. Genetics 192: 1165–1202. 10.1534/genetics.112.145516 23212898 PMC3512134

[16] Hsu J, Weiss EL (2013) Cell cycle regulated interaction of a yeast Hippo kinase and its activator MO25/Hym1. PLoS ONE 8: e78334 10.1371/journal.pone.0078334 24205201 PMC3804511

[17] Nelson B, Kurischko C, Horecka J, Mody M, Nair P, et al (2003) RAM: a conserved signaling network that regulates Ace2p transcriptional activity and polarized morphogenesis. Mol Biol Cell 14: 3782–3803. 12972564 10.1091/mbc.E03-01-0018 PMC196567

[18] Doolin MT, Johnson AL, Johnston LH, Butler G (2001) Overlapping and distinct roles of the duplicated yeast transcription factors Ace2p and Swi5p. Mol Microbiol 40: 422–432. 11309124 10.1046/j.1365-2958.2001.02388.x

[19] Mazanka E, Alexander J, Yeh BJ, Charoenpong P, Lowery DM, et al (2008) The NDR/LATS family kinase Cbk1 directly controls transcriptional asymmetry. PLoS Biol 6: e203 10.1371/journal.pbio.0060203 18715118 PMC2517623

[20] Colman-Lerner A, Chin TE, Brent R (2001) Yeast Cbk1 and Mob2 activate daughter-specific genetic programs to induce asymmetric cell fates. Cell 107: 739–750. 11747810 10.1016/s0092-8674(01)00596-7

[21] Weiss EL, Kurischko C, Zhang C, Shokat K, Drubin DG, et al (2002) The Saccharomyces cerevisiae Mob2p-Cbk1p kinase complex promotes polarized growth and acts with the mitotic exit network to facilitate daughter cell-specific localization of Ace2p transcription factor. J Cell Biol 158: 885–900. 12196508 10.1083/jcb.200203094 PMC2173146

[22] Jansen JM, Wanless AG, Seidel CW, Weiss EL (2009) Cbk1 regulation of the RNA-binding protein Ssd1 integrates cell fate with translational control. Curr Biol 19: 2114–2120. 10.1016/j.cub.2009.10.071 19962308 PMC2805764

[23] Kurischko C, Kim HK, Kuravi VK, Pratzka J, Luca FC (2011) The yeast Cbk1 kinase regulates mRNA localization via the mRNA-binding protein Ssd1. J Cell Biol 192: 583–598. 10.1083/jcb.201011061 21339329 PMC3044126

[24] Hergovich A (2011) MOB control: reviewing a conserved family of kinase regulators. Cell Signal 23: 1433–1440. 10.1016/j.cellsig.2011.04.007 21539912 PMC3398134

[25] Liu G, Young D (2012) Conserved Orb6 phosphorylation sites are essential for polarized cell growth in Schizosaccharomyces pombe. PLoS ONE 7: e37221 10.1371/journal.pone.0037221 22629372 PMC3357421

[26] Chan EH, Nousiainen M, Chalamalasetty RB, Schafer A, Nigg EA, et al (2005) The Ste20-like kinase Mst2 activates the human large tumor suppressor kinase Lats1. Oncogene 24: 2076–2086. 15688006 10.1038/sj.onc.1208445

[27] Stegert MR, Hergovich A, Tamaskovic R, Bichsel SJ, Hemmings BA (2005) Regulation of NDR protein kinase by hydrophobic motif phosphorylation mediated by the mammalian Ste20-like kinase MST3. Mol Cell Biol 25: 11019–11029. 16314523 10.1128/MCB.25.24.11019-11029.2005 PMC1316964

[28] Kannan N, Haste N, Taylor SS, Neuwald AF (2007) The hallmark of AGC kinase functional divergence is its C-terminal tail, a cis-acting regulatory module. Proc Natl Acad Sci U S A 104: 1272–1277. 17227859 10.1073/pnas.0610251104 PMC1783090

[29] Pearce LR, Komander D, Alessi DR (2010) The nuts and bolts of AGC protein kinases. Nat Rev Mol Cell Biol 11: 9–22. 10.1038/nrm2822 20027184

[30] Brace J, Hsu J, Weiss EL (2011) Mitotic exit control of the Saccharomyces cerevisiae Ndr/LATS kinase Cbk1 regulates daughter cell separation after cytokinesis. Mol Cell Biol 31: 721–735. 10.1128/MCB.00403-10 21135117 PMC3028648

[31] Panozzo C, Bourens M, Nowacka A, Herbert CJ (2010) Mutations in the C-terminus of the conserved NDR kinase, Cbk1p of Saccharomyces cerevisiae, make the protein independent of upstream activators. Mol Genet Genomics 283: 111–122. 10.1007/s00438-009-0501-3 19967545

[32] Bichsel SJ, Tamaskovic R, Stegert MR, Hemmings BA (2004) Mechanism of activation of NDR (nuclear Dbf2-related) protein kinase by the hMOB1 protein. J Biol Chem 279: 35228–35235. 15197186 10.1074/jbc.M404542200

[33] Mrkobrada S, Boucher L, Ceccarelli DF, Tyers M, Sicheri F (2006) Structural and functional analysis of Saccharomyces cerevisiae Mob1. J Mol Biol 362: 430–440. 16934835 10.1016/j.jmb.2006.07.007

[34] Stavridi ES, Harris KG, Huyen Y, Bothos J, Verwoerd PM, et al (2003) Crystal structure of a human Mob1 protein: toward understanding Mob-regulated cell cycle pathways. Structure 11: 1163–1170. 12962634 10.1016/s0969-2126(03)00182-5

[35] Tamaskovic R, Bichsel SJ, Hemmings BA (2003) NDR family of AGC kinases—essential regulators of the cell cycle and morphogenesis. FEBS Lett 546: 73–80. 12829239 10.1016/s0014-5793(03)00474-5

[36] Jacobs M, Hayakawa K, Swenson L, Bellon S, Fleming M, et al (2006) The structure of dimeric ROCK I reveals the mechanism for ligand selectivity. J Biol Chem 281: 260–268. 16249185 10.1074/jbc.M508847200

[37] Madhusudan, Trafny EA, Xuong NH, Adams JA, Ten Eyck LF, et al (1994) cAMP-dependent protein kinase: crystallographic insights into substrate recognition and phosphotransfer. Protein Sci 3: 176–187. 8003955 10.1002/pro.5560030203 PMC2142788

[38] Narayana N, Cox S, Shaltiel S, Taylor SS, Xuong N (1997) Crystal structure of a polyhistidine-tagged recombinant catalytic subunit of cAMP-dependent protein kinase complexed with the peptide inhibitor PKI(5–24) and adenosine. Biochemistry 36: 4438–4448. 9109651 10.1021/bi961947+

[39] Yang J, Cron P, Thompson V, Good VM, Hess D, et al (2002) Molecular mechanism for the regulation of protein kinase B/Akt by hydrophobic motif phosphorylation. Mol Cell 9: 1227–1240. 12086620 10.1016/s1097-2765(02)00550-6

[40] Mok J, Kim PM, Lam HY, Piccirillo S, Zhou X, et al (2010) Deciphering protein kinase specificity through large-scale analysis of yeast phosphorylation site motifs. Sci Signal 3: ra12 10.1126/scisignal.2000482 20159853 PMC2846625

[41] Ubersax JA, Ferrell JE Jr (2006) A noisy ‘Start’ to the cell cycle. Mol Syst Biol 2: 2006.0014. 10.1038/msb4100056 PMC1681488 16738559

[42] Davey NE, Van Roey K, Weatheritt RJ, Toedt G, Uyar B, et al (2012) Attributes of short linear motifs. Mol Biosyst 8: 268–281. 10.1039/c1mb05231d 21909575

[43] Remenyi A, Good MC, Lim WA (2006) Docking interactions in protein kinase and phosphatase networks. Curr Opin Struct Biol 16: 676–685. 17079133 10.1016/j.sbi.2006.10.008

[44] Nguyen Ba AN, Yeh BJ, van Dyk D, Davidson AR, Andrews BJ, et al (2012) Proteome-wide discovery of evolutionary conserved sequences in disordered regions. Sci Signal 5: rs1 10.1126/scisignal.2002515 22416277 PMC4876815

[45] Endicott JA, Noble ME, Johnson LN (2012) The structural basis for control of eukaryotic protein kinases. Annu Rev Biochem 81: 587–613. 10.1146/annurev-biochem-052410-090317 22482904

[46] Morris GM, Huey R, Lindstrom W, Sanner MF, Belew RK, et al (2009) AutoDock4 and AutoDockTools4: automated docking with selective receptor flexibility. J Comput Chem 30: 2785–2791. 10.1002/jcc.21256 19399780 PMC2760638

[47] Jansen JM, Barry MF, Yoo CK, Weiss EL (2006) Phosphoregulation of Cbk1 is critical for RAM network control of transcription and morphogenesis. J Cell Biol 175: 755–766. 17145962 10.1083/jcb.200604107 PMC2064675

[48] Lai AC, Nguyen Ba AN, Moses AM (2012) Predicting kinase substrates using conservation of local motif density. Bioinformatics 28: 962–969. 10.1093/bioinformatics/bts060 22302575

[49] Chatr-Aryamontri A, Breitkreutz BJ, Heinicke S, Boucher L, Winter A, et al (2013) The BioGRID interaction database: 2013 update. Nucleic Acids Res 41: D816–D823. 10.1093/nar/gks1158 23203989 PMC3531226

[50] Sadowski I, Breitkreutz BJ, Stark C, Su TC, Dahabieh M, et al (2013) The PhosphoGRID Saccharomyces cerevisiae protein phosphorylation site database: version 2.0 update. Database (Oxford) 2013: bat026. 23674503 10.1093/database/bat026 PMC3653121

[51] Yang J, Cron P, Good VM, Thompson V, Hemmings BA, et al (2002) Crystal structure of an activated Akt/protein kinase B ternary complex with GSK3-peptide and AMP-PNP. Nat Struct Biol 9: 940–944. 12434148 10.1038/nsb870

[52] Cook D, Hoa LY, Gomez V, Gomez M, Hergovich A (2014) Constitutively active NDR1-PIF kinase functions independent of MST1 and hMOB1 signalling. Cell Signal 26: 1657–1667. 10.1016/j.cellsig.2014.04.011 24747552 PMC4049220

[53] Malakhova M, Kurinov I, Liu K, Zheng D, D’Angelo I, et al (2009) Structural diversity of the active N-terminal kinase domain of p90 ribosomal S6 kinase 2. PLoS ONE 4: e8044 10.1371/journal.pone.0008044 19956600 PMC2779450

[54] Jeffrey PD, Russo AA, Polyak K, Gibbs E, Hurwitz J, et al (1995) Mechanism of CDK activation revealed by the structure of a cyclinA-CDK2 complex. Nature 376: 313–320. 7630397 10.1038/376313a0

[55] Garai A, Zeke A, Gogl G, Toro I, Fordos F, et al (2012) Specificity of linear motifs that bind to a common mitogen-activated protein kinase docking groove. Sci Signal 5: ra74 10.1126/scisignal.2003004 23047924 PMC3500698

[56] Lee T, Hoofnagle AN, Kabuyama Y, Stroud J, Min X, et al (2004) Docking motif interactions in MAP kinases revealed by hydrogen exchange mass spectrometry. Mol Cell 14: 43–55. 15068802 10.1016/s1097-2765(04)00161-3

[57] Bender L, Lo HS, Lee H, Kokojan V, Peterson V, et al (1996) Associations among PH and SH3 domain-containing proteins and Rho-type GTPases in Yeast. J Cell Biol 133: 879–894. 8666672 10.1083/jcb.133.4.879 PMC2120828

[58] Matsui Y, Matsui R, Akada R, Toh-e A (1996) Yeast src homology region 3 domain-binding proteins involved in bud formation. J Cell Biol 133: 865–878. 8666671 10.1083/jcb.133.4.865 PMC2120841

[59] Norden C, Mendoza M, Dobbelaere J, Kotwaliwale CV, Biggins S, et al (2006) The NoCut pathway links completion of cytokinesis to spindle midzone function to prevent chromosome breakage. Cell 125: 85–98. 16615892 10.1016/j.cell.2006.01.045

[60] Shepard KA, Gerber AP, Jambhekar A, Takizawa PA, Brown PO, et al (2003) Widespread cytoplasmic mRNA transport in yeast: identification of 22 bud-localized transcripts using DNA microarray analysis. Proc Natl Acad Sci U S A 100: 11429–11434. 13679573 10.1073/pnas.2033246100 PMC208774

[61] Bourens M, Panozzo C, Nowacka A, Imbeaud S, Mucchielli MH, et al (2009) Mutations in the Saccharomyces cerevisiae kinase Cbk1p lead to a fertility defect that can be suppressed by the absence of Brr1p or Mpt5p (Puf5p), proteins involved in RNA metabolism. Genetics 183: 161–173. 10.1534/genetics.109.105130 19546315 PMC2746141

[62] Goldstrohm AC, Seay DJ, Hook BA, Wickens M (2007) PUF protein-mediated deadenylation is catalyzed by Ccr4p. J Biol Chem 282: 109–114. 17090538 10.1074/jbc.M609413200

[63] Tompa P, Davey NE, Gibson TJ, Babu MM (2014) A million peptide motifs for the molecular biologist. Mol Cell 55: 161–169. 10.1016/j.molcel.2014.05.032 25038412

[64] Jin J, Pawson T (2012) Modular evolution of phosphorylation-based signalling systems. Philos Trans R Soc Lond B Biol Sci 367: 2540–2555. 10.1098/rstb.2012.0106 22889906 PMC3415845

[65] Richter DJ, King N (2013) The genomic and cellular foundations of animal origins. Annu Rev Genet 47: 509–537. 10.1146/annurev-genet-111212-133456 24050174

[66] Kabsch W (2010) XDS. Acta Crystallogr D Biol Crystallogr 66: 125–132. 10.1107/S0907444909047337 20124692 PMC2815665

[67] McCoy AJ, Grosse-Kunstleve RW, Adams PD, Winn MD, Storoni LC, et al (2007) Phaser crystallographic software. J Appl Crystallogr 40: 658–674. 19461840 10.1107/S0021889807021206 PMC2483472

[68] Adams PD, Afonine PV, Bunkoczi G, Chen VB, Davis IW, et al (2010) PHENIX: a comprehensive Python-based system for macromolecular structure solution. Acta Crystallogr D Biol Crystallogr 66: 213–221. 10.1107/S0907444909052925 20124702 PMC2815670

[69] Emsley P, Lohkamp B, Scott WG, Cowtan K (2010) Features and development of Coot. Acta Crystallogr D Biol Crystallogr 66: 486–501. 10.1107/S0907444910007493 20383002 PMC2852313

[70] Pronk S, Pall S, Schulz R, Larsson P, Bjelkmar P, et al (2013) GROMACS 4.5: a high-throughput and highly parallel open source molecular simulation toolkit. Bioinformatics 29: 845–854. 10.1093/bioinformatics/btt055 23407358 PMC3605599

[71] Duan Y, Wu C, Chowdhury S, Lee MC, Xiong G, et al (2003) A point-charge force field for molecular mechanics simulations of proteins based on condensed-phase quantum mechanical calculations. J Comput Chem 24: 1999–2012. 14531054 10.1002/jcc.10349

[72] Jorgensen WL, Chandrasekhar J, Madura JD, Impey RW, Klein ML (1983) Comparison of simple potential functions for simulating liquid water. J Chem Phys 79: 926–935.

[73] Hess B, Bekker H, Berendsen HJC, Fraaije JGEM (1998) LINCS: A Linear Constraint Solver for Molecular Simulations. J Comput Chem 18: 1463–1472.

[74] Bussi G, Donadio D, Parrinello M (2007) Canonical sampling through velocity rescaling. J Chem Phys 126: 014101 17212484 10.1063/1.2408420

[75] Ren P, Ponder JW (2003) Polarizable atomic multipole water model for molecular mechanics simulation. J Phys Chem B 107: 5933–5947.

[76] Wang J, Cieplak P, Kollman PA (2000) How well does a restrained electrostatic potential (RESP) model perform in calculating conformational energies of organic and biological molecules? J Comput Chem 21: 1049–1074.

[77] Hess B, Kutzner C, van der Spoel D, Lindahl E (2008) GROMACS 4: algorithms for highly efficient, load-balanced, and scalable molecular simulation. J Chem Theory Comput 4: 435–447. 26620784 10.1021/ct700301q

[78] Hetenyi C, van der Spoel D (2002) Efficient docking of peptides to proteins without prior knowledge of the binding site. Protein Sci 11: 1729–1737. 12070326 10.1110/ps.0202302 PMC2373668

[79] Hetenyi C, van der Spoel D (2006) Blind docking of drug-sized compounds to proteins with up to a thousand residues. FEBS Lett 580: 1447–1450. 16460734 10.1016/j.febslet.2006.01.074

[80] Hetenyi C, van der Spoel D (2011) Toward prediction of functional protein pockets using blind docking and pocket search algorithms. Protein Sci 20: 880–893. 10.1002/pro.618 21413095 PMC3125872

[81] Byrne KP, Wolfe KH (2005) The Yeast Gene Order Browser: combining curated homology and syntenic context reveals gene fate in polyploid species. Genome Res 15: 1456–1461. 16169922 10.1101/gr.3672305 PMC1240090

