# Supplementary figures and images for: The Structure of an NDR/LATS Kinase–Mob Complex Reveals a Novel Kinase–Coactivator System and Substrate Docking Mechanism
Source: PLoS Biol. 2015 May 12;13(5):e1002146. doi: 10.1371/journal.pbio.1002146 (PMC4428629; doi:10.1371/journal.pbio.1002146)

Figure S1. Multiple sequence alignment of Ndr kinases.

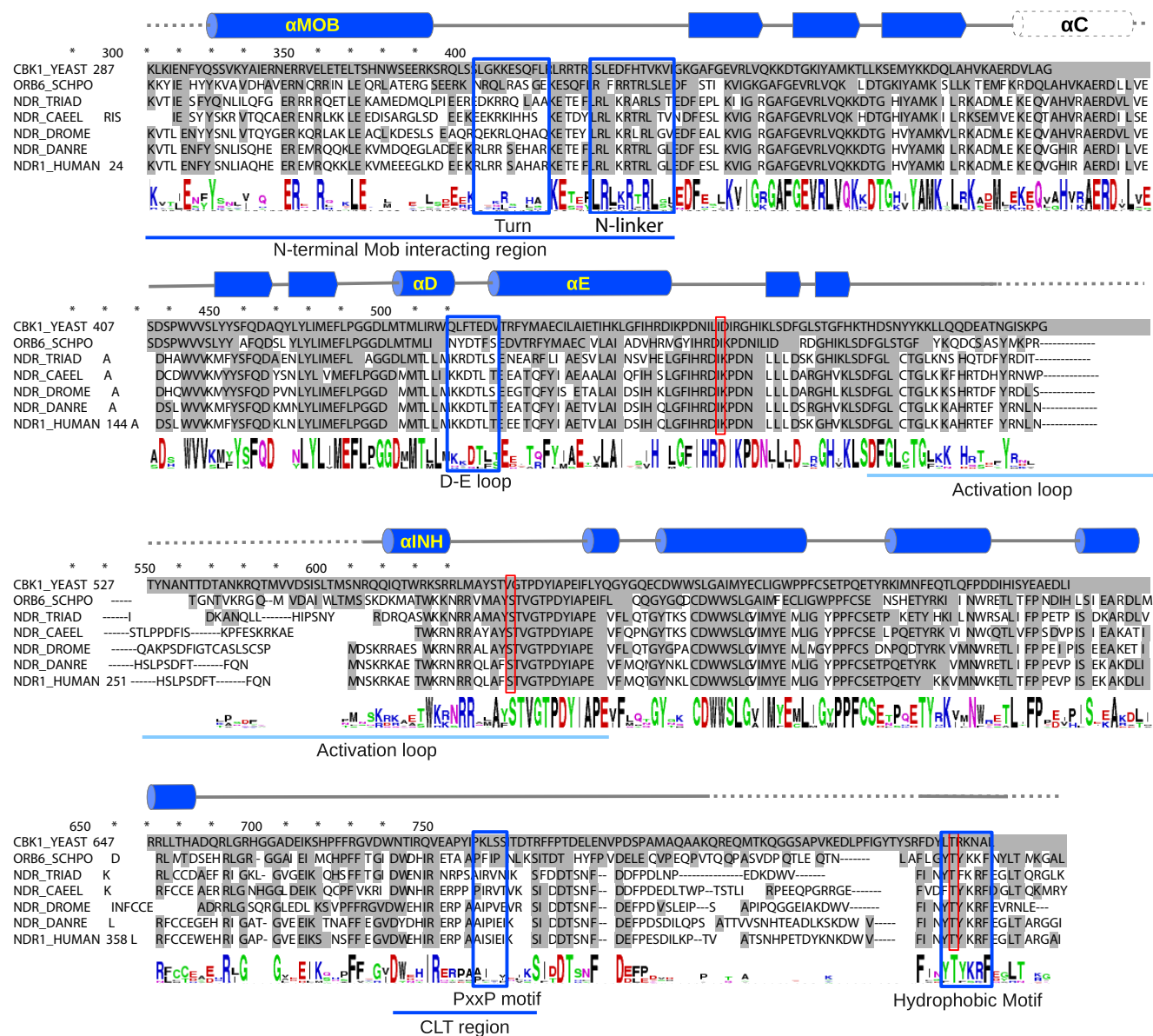

Supplement: S1 Fig — Sequence comparison of Cbk1 from Saccharomyces cerevisae (CBK1_YEAST) with other NDR kinases from different organisms (SCHPO: Schizosaccharomyces pombe; TRIAD: Trichoplax adherens; CAEEL: Caenorhabditis elegans; DROME: Drosphila melanogaster; DANRE: Danio rerio). The consensus sequence is presented as a sequence logo. Residues and motifs important for Cbk1 activity or for its regulation are boxed: Asp475 is the catalytic aspartate, Ser570 is an autophosphorylation site, while Thr743 is phosphorylated by an upstream kinase. Important regions are underlined. Highlighting is based on sequence identity compared to Cbk1. Secondary structure elements from the crystallographic models are shown above the sequences. Dashed lines indicate regions that could not be built into the crystallographic model of the Cbk1–Mob2 complex. (PDF) [file pbio.1002146.s007.pdf]

Figure S2. Additional information on the crystal structures of Cbk1–Mob2 complexes

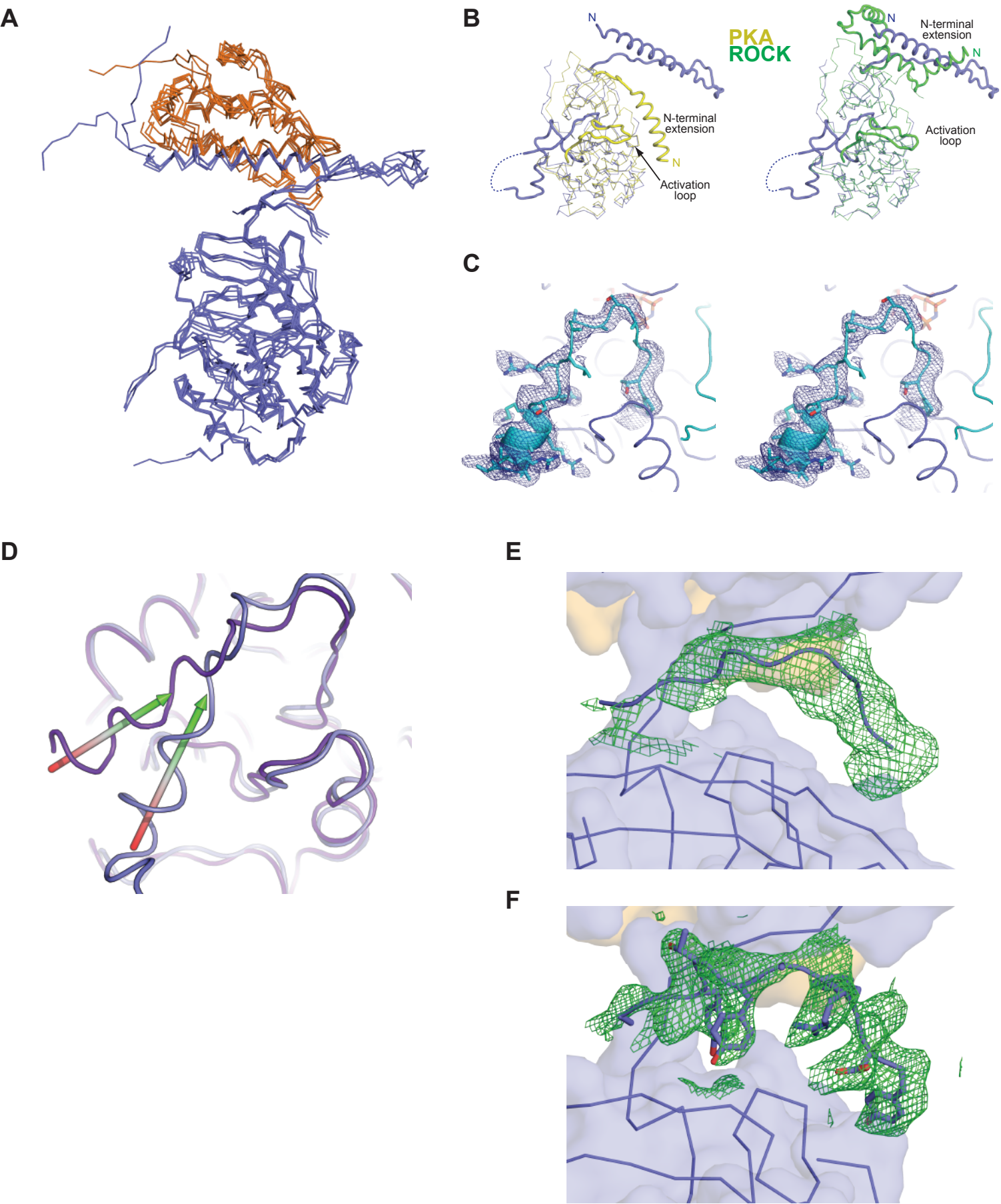

Supplement: S2 Fig — (A) Superimposition of the crystallographic models for Cbk1–Mob2 complexes. All four complexes (from three different crystal forms) display the same Cbk1 and Mob2 domain arrangements. (B) The Cbk1 kinase domain (shown in blue) is similar to the AGC kinase domains from related kinases such as PKA (PDB ID: 1JLU; yellow) and ROCK1 (PDB ID: 2ETR; green) [36,37]. In contrast, the activation loop and N-terminal kinase domain extensions adopt markedly different structures. (AGC kinase domains are shown in thin ribbon, while activation loops and N-terminal kinase domain extensions are shown in thicker tube representation.) (C) Stereo view of the final 2Fo-Fc electron density map around the activation loop (cyan) at 3.3 Å resolution. (D) In the two different crystal forms of Cbk1(T743E), the αINH is rotated with an angle of ~30° due to different crystal packing. For clarity, Cbk1 from crystal form A is colored purple and from crystal form B is colored in blue. (E) Simulated annealing 2Fo-Fc omit map contoured at 1σ and calculated around the HM motif region for the 4.5 Å resolution Cbk1–Mob2 structure. (F) Omit map (generated with the same protocol) for the 3.3 Å resolution Cbk1–Mob2 crystal structure. (PDF) [file pbio.1002146.s008.pdf]

A

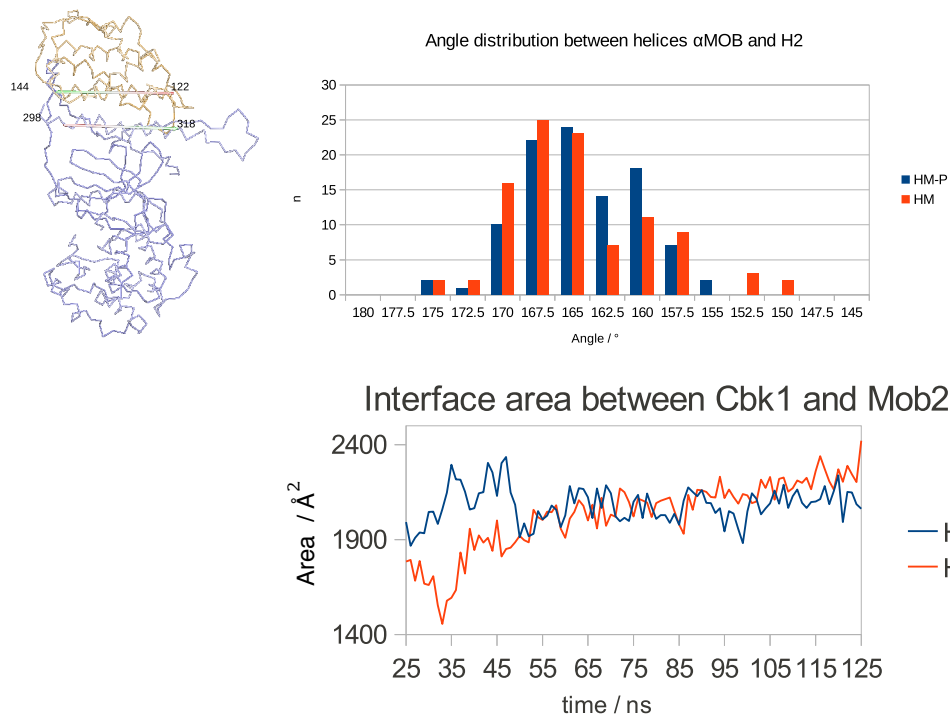

B

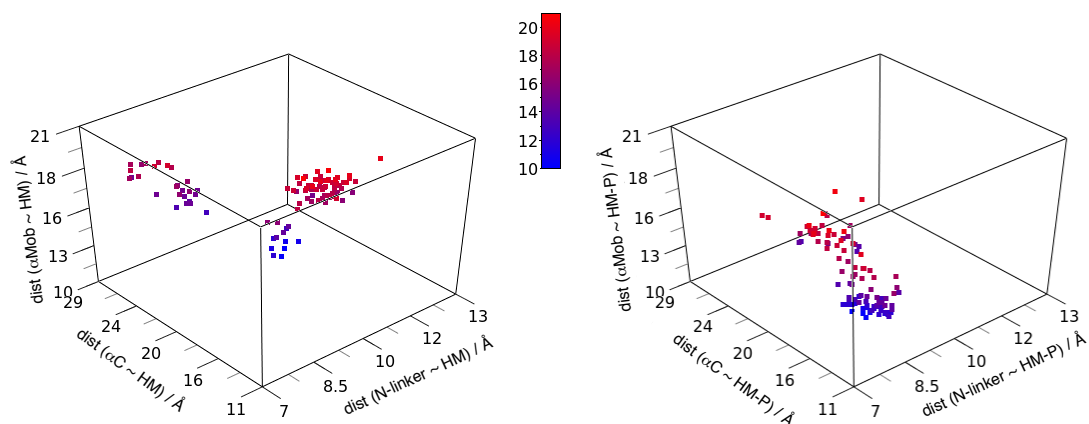

C

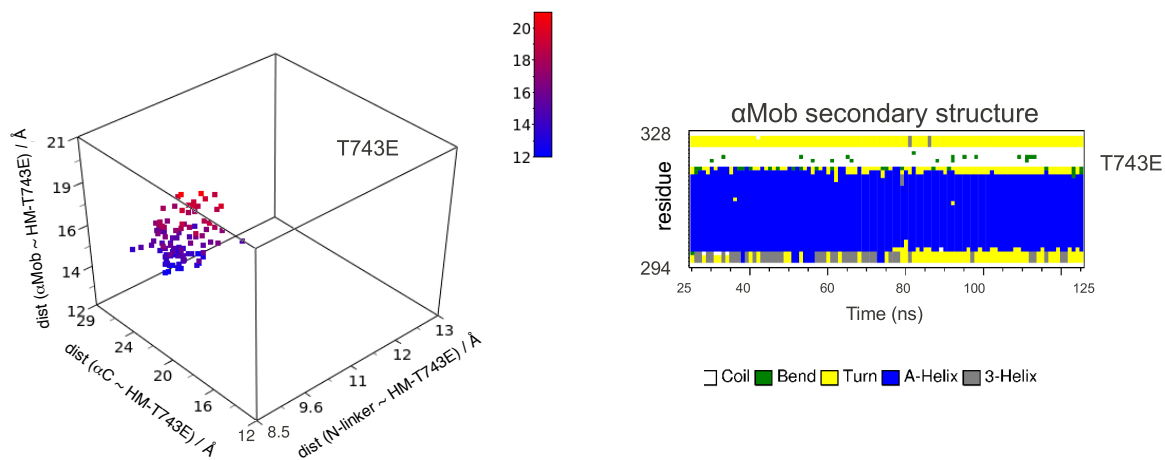

Figure S4

Supplement: S4 Fig — (A) Cbk1–Mob2 interface analysis. Upper panel shows the angle distribution between αMOB (Cbk1) and H2 (Mob2) as defined on the left panel. The small differences between the HM-P complex and the HM complex are not statistically significant. Lower panel displays interface area between Cbk1 and Mob2. These analyses show that there is no major global change at the Cbk1–Mob2 interface during MD, confirming that the changes shown in Fig 3 occur because HM-mediated local interactions change within the binding slot. (B) Three-dimensional N-linker–HM, αC–HM and αMOB–HM distance scatter plots on Cbk1 with unphosphorylated and phosphorylated HM. MD simulations were identical to those on Cbk1–Mob2 complexes, but the Mob2 protein chain was removed from the starting MD model. (C) Three-dimensional N-linker–HM, αC–HM, and αMOB–HM distance scatter plots on Cbk1 with HM-E. MD simulations were identical to those on Cbk1–Mob2 complexes, but Cbk1 T743 was mutated to glutamic acid. MD data can be found in S1 Data. (PDF) [file pbio.1002146.s010.pdf]

Figure S6. Cbk1-Ace2 docking confers robustness to regulation of Ace2 target gene transcription

**A**

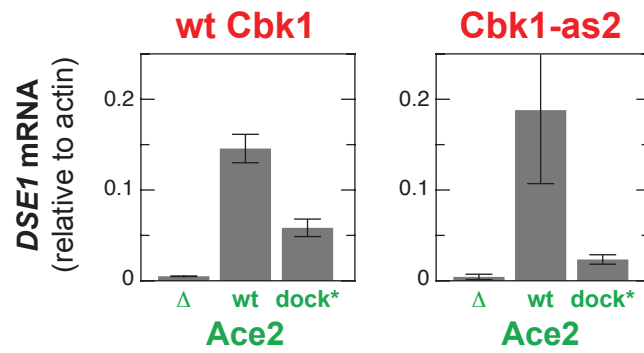

**B**

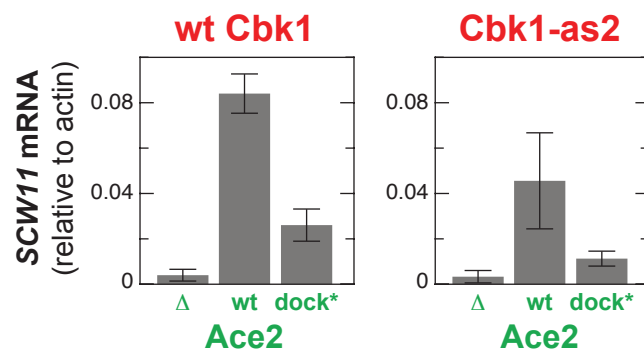

Supplement: S6 Fig — (A) CBK1 WT cells carrying ace2 dock* have no significant reduction in transcript levels of the DSE1 gene, while cbk1-as2 cells carrying ace2 dock* exhibit dramatically reduced DSE1 transcription. (B) CBK1 WT cells carrying ace2 dock* exhibit modest reduction in transcript levels of the SCW11 gene, while cbk1-as2 cells carrying ace2 dock* exhibit strongly reduced SCW11 transcription. Transcription data can be found in S5 Data. (PDF) [file pbio.1002146.s012.pdf]

Figure S8. Comparative analysis of Cbk1 regions involved in docking motif binding

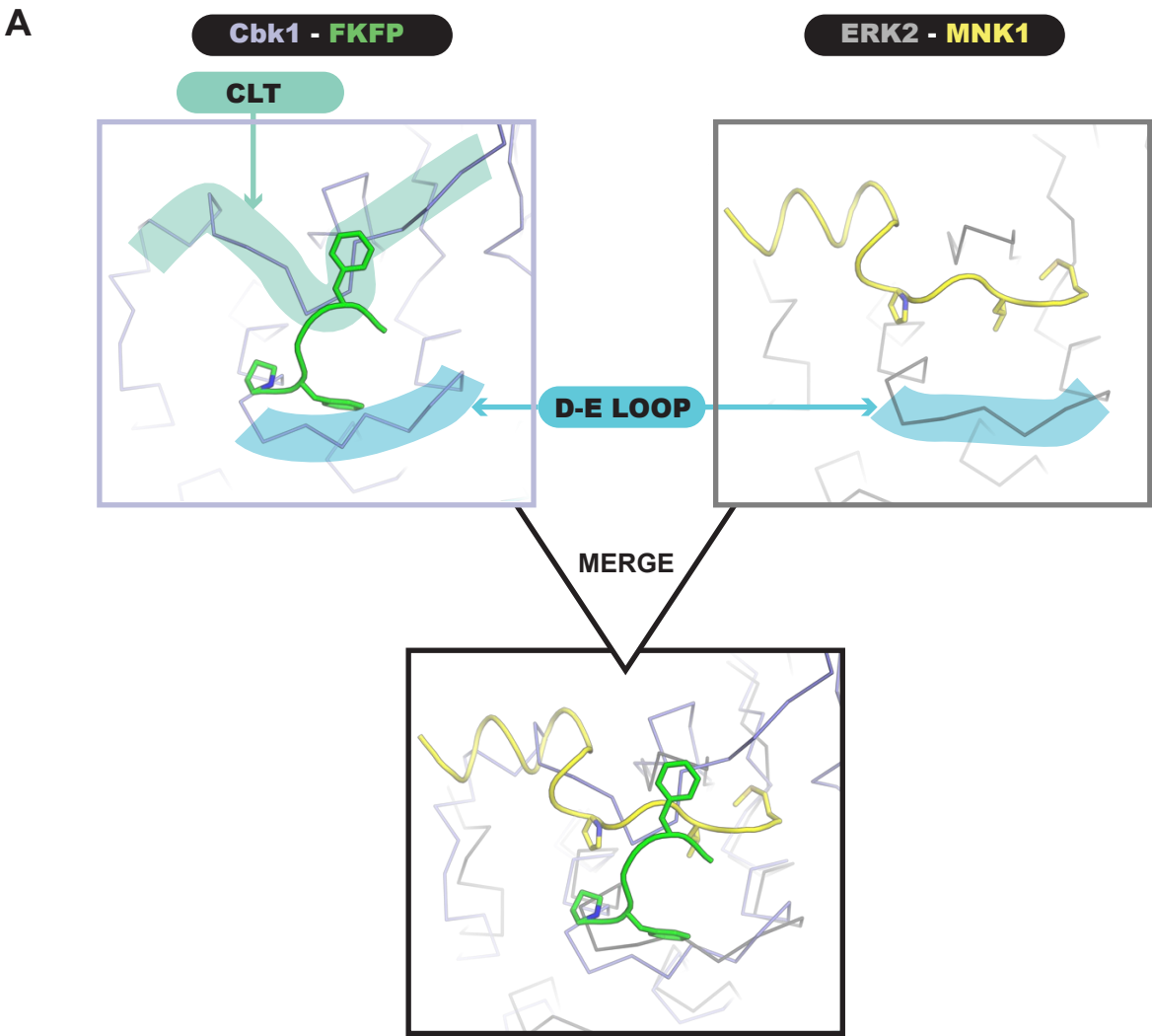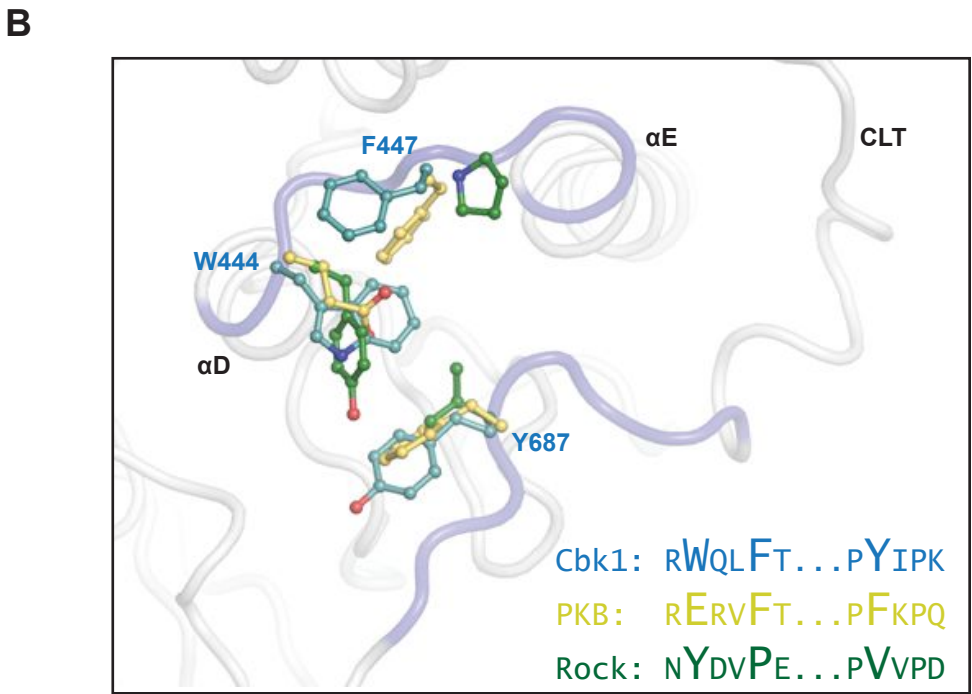

Supplement: S8 Fig — (A) A comparison of Cbk1 bound to the FKFP core docking motif and ERK2 bound to the MNK1 docking motif (2Y9IQ) [55]. The loop between kinase domain D and E helices is noted for both Cbk1 and ERK2, and Cbk1’s CLT region is also highlighted. An overlay of the structures shows different peptide binding modes on roughly corresponding surface regions of both kinases. (B) Overlay of the Cbk1 docking region with the corresponding surface of PKA (PDB ID: 1JLU; yellow) and ROCK1 (PDB ID: 2ETR; green) [36,37]. Side chains of amino acids differ, but are placed in a roughly similar geometry. (PDF) [file pbio.1002146.s014.pdf]

**C**

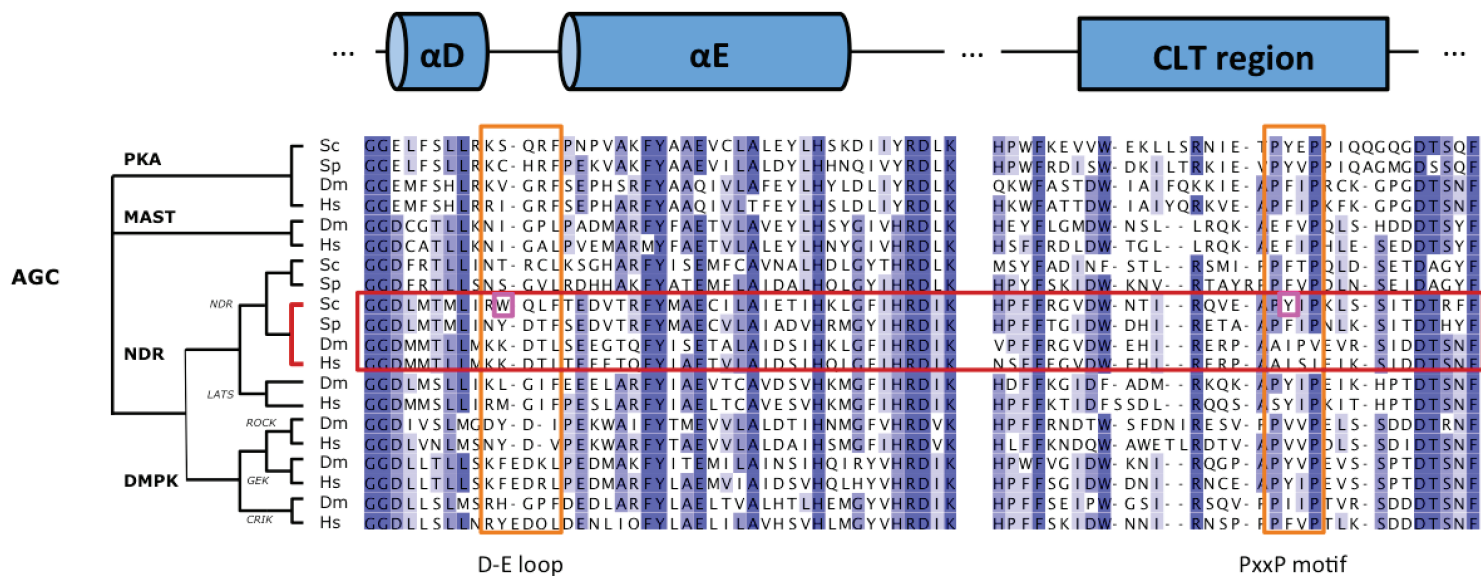

Supplement: S9 Fig — A gene tree of AGC kinases related to the NDR family, with the closest Cbk1 orthologs in S. cerevisiae (Sc), Sc. pombe (Sp), D. melanogaster (Dm), and Homo sapiens (Hs) bracketed in red. The loop between the D and E helices in the kinase C-lobe and the PxxP motif in the CLT region are enclosed in orange boxes. Bulky hydrophobic amino acids involved in docking motif binding in Cbk1 are boxed in pink. Tree is not drawn to scale. PKA and MAST are used as outgroups to the NDR family. (PDF) [file pbio.1002146.s015.pdf]
