# Supplementary material for: The Structure of an NDR/LATS Kinase–Mob Complex Reveals a Novel Kinase–Coactivator System and Substrate Docking Mechanism
Source: PLoS Biol. 2015 May 12;13(5):e1002146. doi: 10.1371/journal.pbio.1002146 (PMC4428629; doi:10.1371/journal.pbio.1002146)

**Figure S3. Characterization of the Cbk1 hydrophobic motif and its binding to the Cbk1–Mob2 complex**

**A**

| | Direct $K_d$ (μM) | Competitive $K_d$ with HM-P (μM) | Competitive $K_d$ with pepSSD1 (μM) |
|---|---|---|---|
| HM peptide | 33 | ND | ND |
| HM-P peptide | 6 | 45 | No competitive binding |

**B**

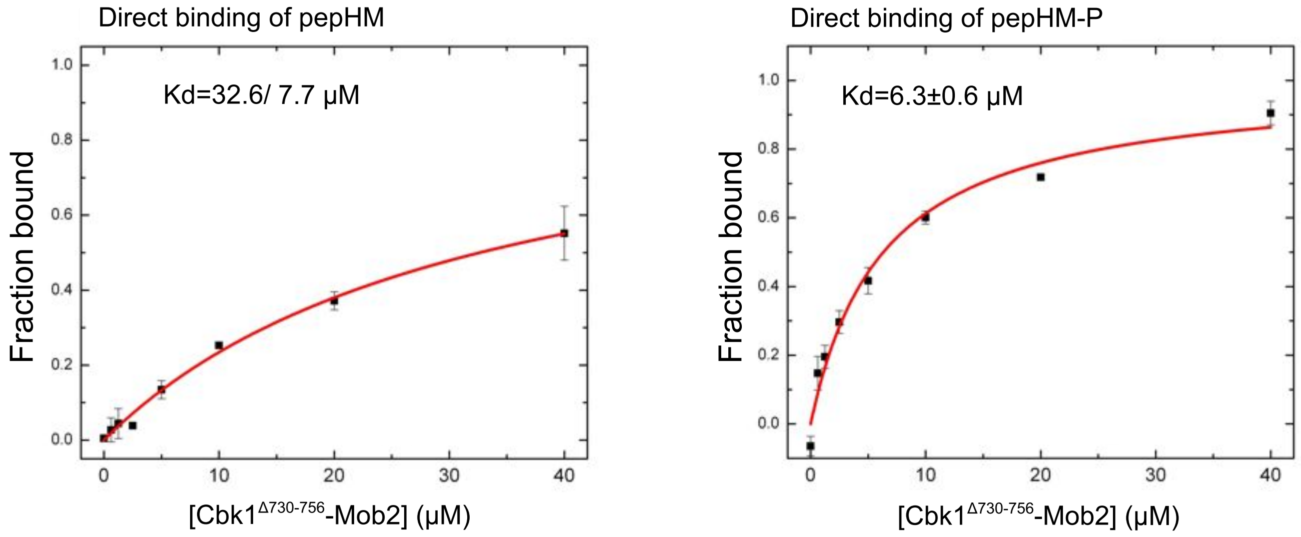


**C**

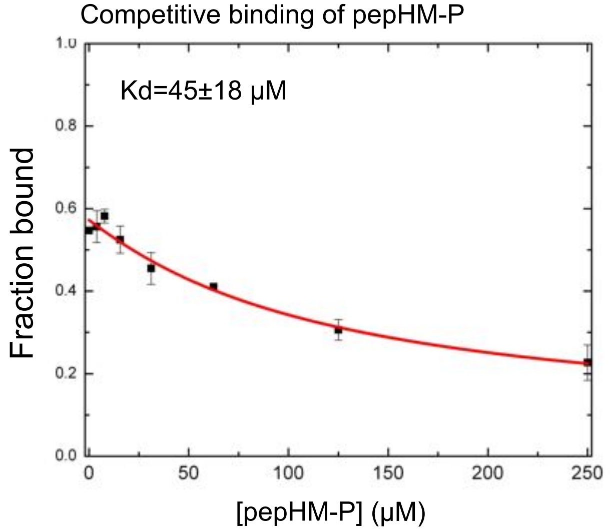


**D**

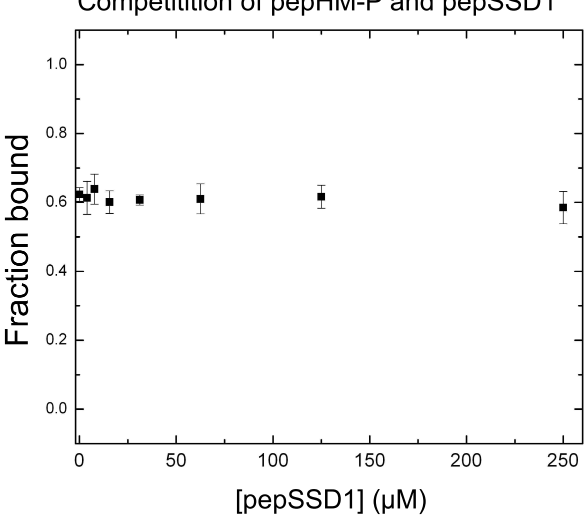

Supplement: S3 Fig — (A) Summary of the FP binding experiments with Cbk1-HM-containing peptides. Although the Cbk1–Mob2 and the Cbk1(T743E)–Mob2 complexes did not show major changes in the position of their Cbk1 HM motifs, we compared the binding affinity of peptides containing unphosphorylated and phosphorylated HM motifs to a Cbk1∆730–756 variant complexed with Mob2 (which lacks the HM region) in trans. These in vitro binding affinity measurements indicated that the phosphorylated HM peptide bound more than 5-fold stronger in trans into the open Cbk1 HM-binding slot. Binding of a carboxyfluorescein-labeled Cbk1-HM-containing peptide was monitored and compared to the binding of the phosphorylated HM peptide. In direct titration binding experiments, the binding of the labeled peptide was monitored and binding isotherms were fit to a classical binding equation. Competitive titration experiments (starting from 60%–80% complex formation between the protein and the labeled peptide) monitored the binding of unlabeled peptide as it competed with the labeled peptide for the same binding site. Competitive titrations can indirectly report on the binding affinity of unlabeled peptides if data are fit to a competition binding equation. ND, not determined. (Note that these experiments monitored the binding of peptides in trans, and they do not report on the binding of the HM motif as part of the full-length Cbk1.) (B) Phosphorylated HM motif binds to Cbk1Δ730–756–Mob2 with increased affinity. (C) Competitive titration curves for monitoring HM and HM-P peptide binding to the Cbk1Δ730–756–Mob2 construct. The markedly reduced binding affinity for the unlabeled versus the labeled HM-P peptide is likely due to the presence of the fluorophore, which seems to artificially increase binding. (D) Competitive titration binding curve for monitoring labeled HM-P and Ssd1 docking peptide binding. This panel shows that the docking peptide could not compete with the HM peptide, indicating that the Ssd1 docking pep [file pbio.1002146.s009.pdf]
