# Supplementary material for: The Structure of an NDR/LATS Kinase–Mob Complex Reveals a Novel Kinase–Coactivator System and Substrate Docking Mechanism
Source: PLoS Biol. 2015 May 12;13(5):e1002146. doi: 10.1371/journal.pbio.1002146 (PMC4428629; doi:10.1371/journal.pbio.1002146)

Figure S5. Identification and analysis of docking motifs in Ace2 and Ssd1

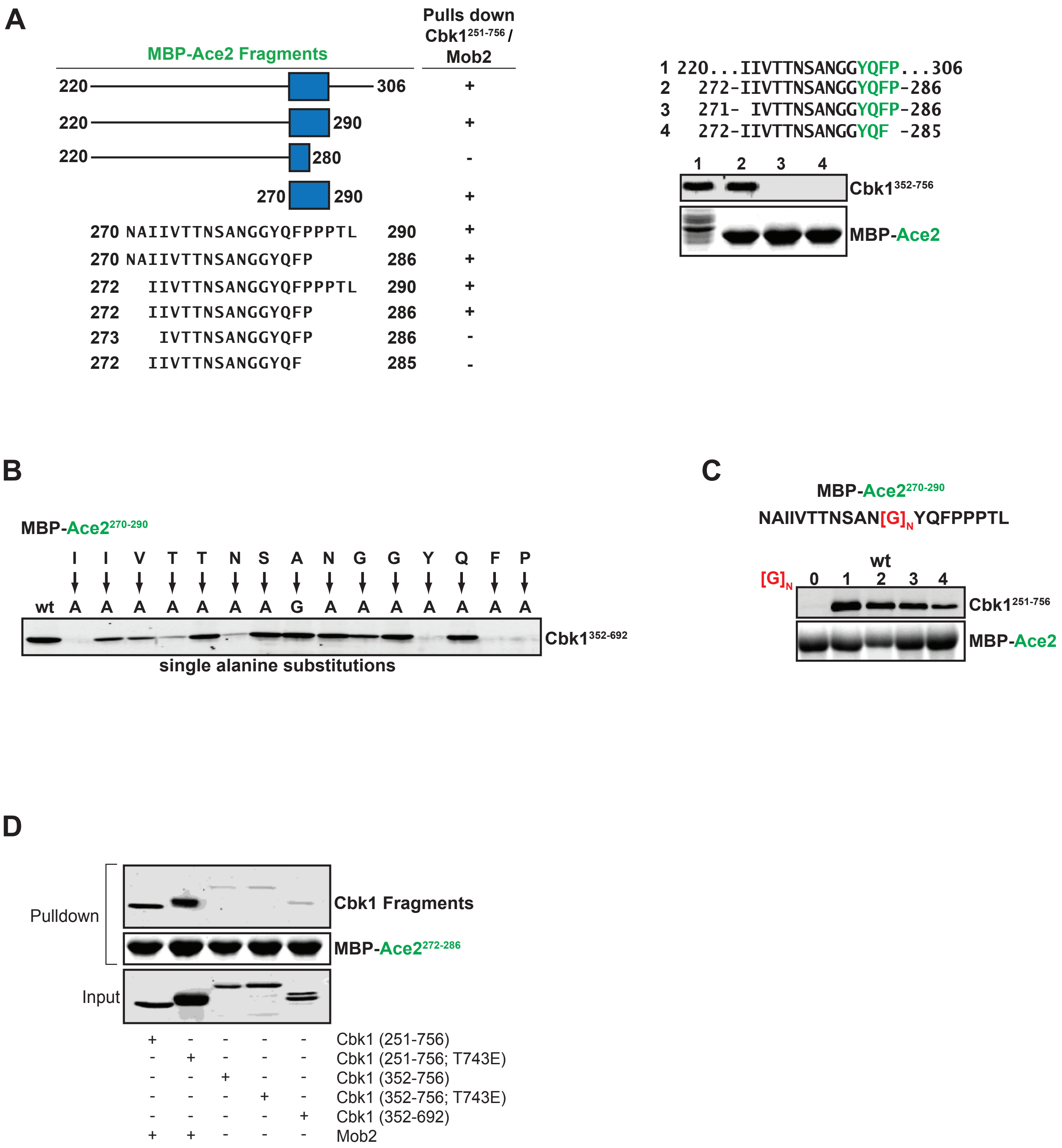

Figure S5. Identification and analysis of docking motifs in Ace2 and Ssd1 (continued)

E

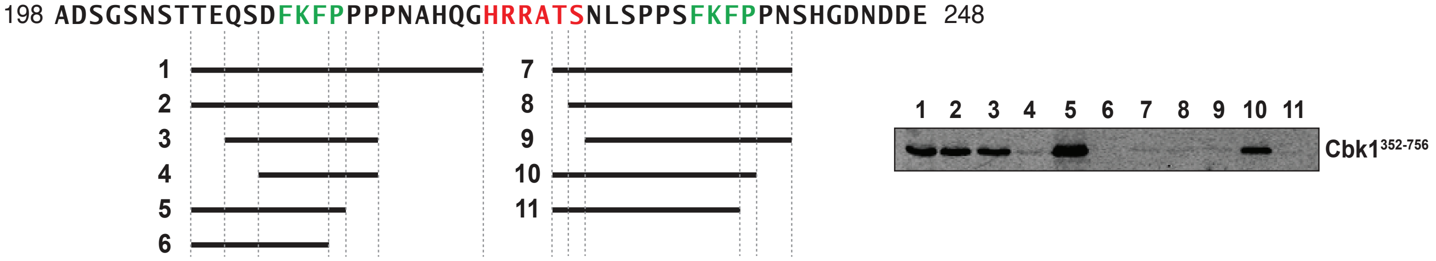

F

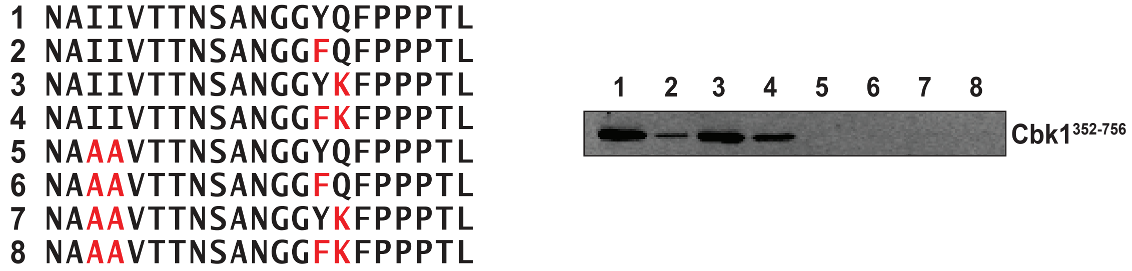

G

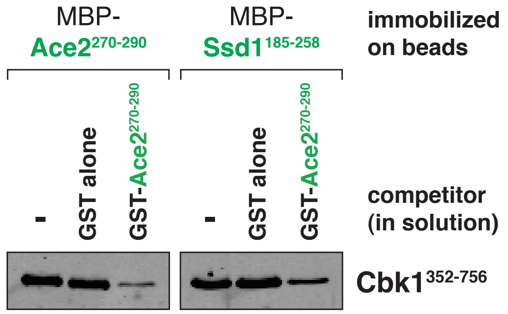

Figure S5. Identification and analysis of docking motifs in Ace2 and Ssd1 (continued)

H. Fluorescence polarization (FP) of Ssd1 (top) and Ace2 (bottom) docking motif peptides with Cbk1-Mob2

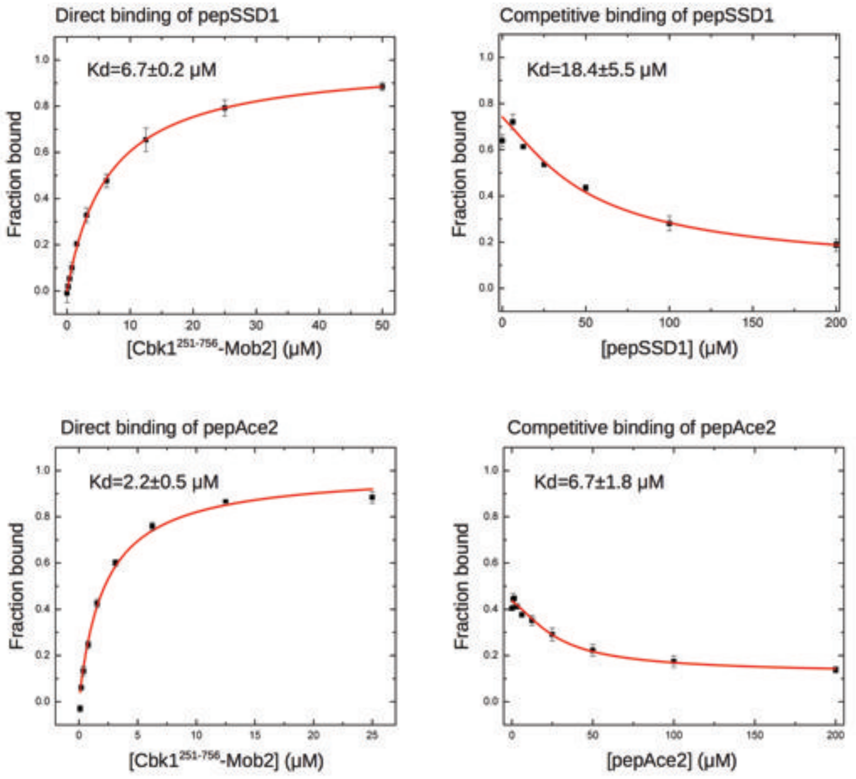

I. FP of Ssd1 with full length Cbk1 kinase domain (top) or with the HM deleted (bottom). Neither Cbk1 truncation exhibits defects in docking motif interactions.

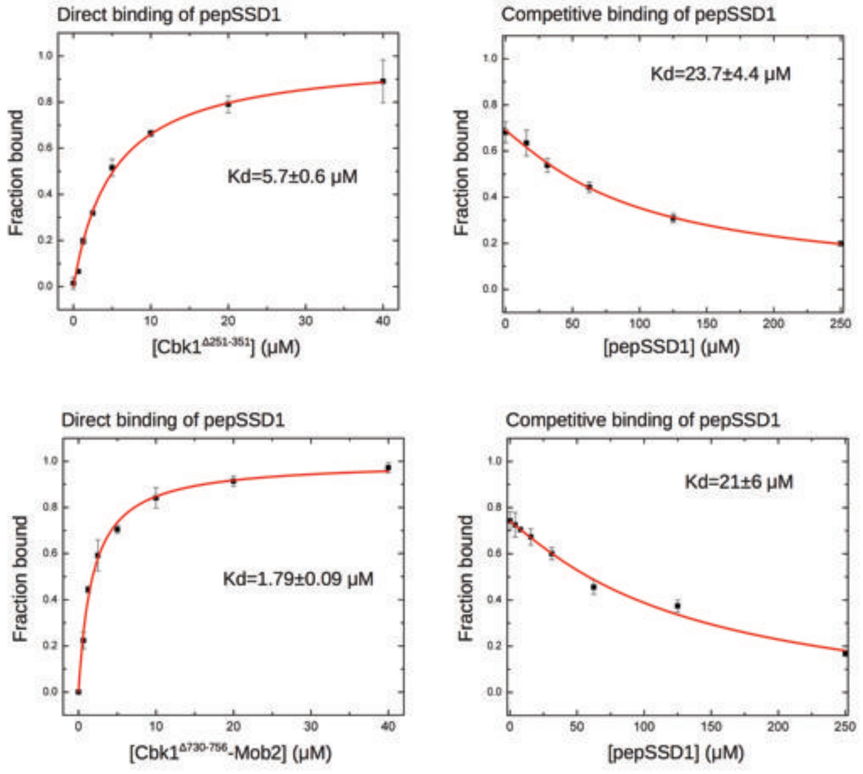

Supplement: S5 Fig — (A) Pulldown of Cbk1–Mob2 by Ace2 truncation fragments (left) and Ace2 fragments centered on the docking motif (right). Smaller fragments than Ace2272–286 abrogate Cbk1 interaction. (B) Alanine scan of Ace2270–290 pulldown with the Cbk1352–692 kinase domain alone highlights the importance of N-terminal hydrophobic residues in addition to the C-terminal core motif. (C) Flexibility analysis of Ace2270–290 by glycine insertion/deletion and pulldown of Cbk1–Mob2. The bipartite motif can be extended, but deletion abrogates Cbk1 interaction. WT Ace2 contains two glycine residues. (D) Cbk1352–756 kinase domain in vitro kinase assay with Ace2102–306. The presence of the docking motif enhances phosphorylation 100-fold as well as enhances Cbk1 autophosphorylation. (E) Pulldown of Cbk1352–756 by Ssd1 truncation fragments containing the N-terminal (1–6) or C-terminal (7–11) docking motif. (F) Pulldown of Cbk1352–756 by the Ace2 docking motif with stepwise conversion to the Ssd1 docking motif. Conversion of the core motif (YQFP → FKFP) could not rescue mutation of N-terminal residues, highlighting the importance of surrounding sequence to the core motif. (G) Competition of Ace2 (left) and Ssd1 (right) docking motifs with unbound Ace2270–290. Competition was analyzed by Cbk1352–756 pulldown. (H) FP affinity measurements of peptides containing Ssd1 (top) and Ace2 (bottom) docking motifs with Cbk1–Mob2. (I) FP of Ssd1 with the Cbk1 kinase domain (top) or with the HM deleted (bottom). Neither Cbk1 truncation exhibited defects in docking motif interactions. FP data can be found in S2 Data. (PDF) [file pbio.1002146.s011.pdf]
