# Supplementary material for: The Structure of an NDR/LATS Kinase–Mob Complex Reveals a Novel Kinase–Coactivator System and Substrate Docking Mechanism
Source: PLoS Biol. 2015 May 12;13(5):e1002146. doi: 10.1371/journal.pbio.1002146 (PMC4428629; doi:10.1371/journal.pbio.1002146)
Supplement: S7 Fig — (A) In proteins that contain matches to the Cbk1 docking motif [YF]xFP, there is a statistically significant enrichment of NDR/LATS phosphorylation consensus sites Hx[RK]xx[ST] compared to proteins that do not contain docking motifs (0.45 versus 0.36 matches per 1,000 amino acids; Fisher’s exact test, p = 0.016). Similar results are obtained for phosphorylation consensus sites for which there is mass spectrometric evidence of phosphorylation as annotated in 2013 by PhosphoGrid [50] (0.040 versus 0.017 phosphorylated matches per 1,000 amino acids; Fisher’s exact test, p = 0.013). If we consider the 50 most conserved phosphorylation consensus sites as identified by ConDens [48], the enrichment is much stronger (0.067 versus 0.011 conserved matches per 1,000 amino acids; Fisher’s exact test, p = 3.629 × 10^−8^). Since many matches to the docking motif ([YF]xFP) might appear in proteins by chance, we also considered proteins that contain conserved docking motifs. In these proteins the enrichments are much stronger. For example, phosphorylation consensus matches are now >8× more like to appear (2.95 versus 0.36 matches per 1,000 amino acids; Fisher’s exact test, p < 10 × 10−10). Similarly, phosphorylated consensus matches and the 50 most conserved phosphorylation consensus matches are >30× and >100× more likely to appear (Fisher’s exact test, p < 10 × 10−6 and p < 10 × 10−10, respectively). These tests show that a conserved docking site makes consensus sites (and conserved consensus sites) more likely to appear, and that the phospho-acceptor residues within these consensus sites are significantly more likely to be among the set of phosphorylated positions annotated in the PhosphoGrid database. (B) In the 11 proteins with conserved [YF]xFP docking motifs, 14/27 (51.8%) of the Cbk1 phosphorylation consensus matches are in the 50 most conserved, which is much more than 50/887 (5.6%) in the proteome (Fisher’s exact test, p < 10 × 10−10) Similarly, consensus matches are more li [file pbio.1002146.s013.pdf]

Figure S7. Co-occurrence of docking motifs and Cbk1 phosphorylation consensus sites is extremely significant, and docking sites predict consensus site conservation and phosphorylation

A.

docking motifs predict phosphorylation sites

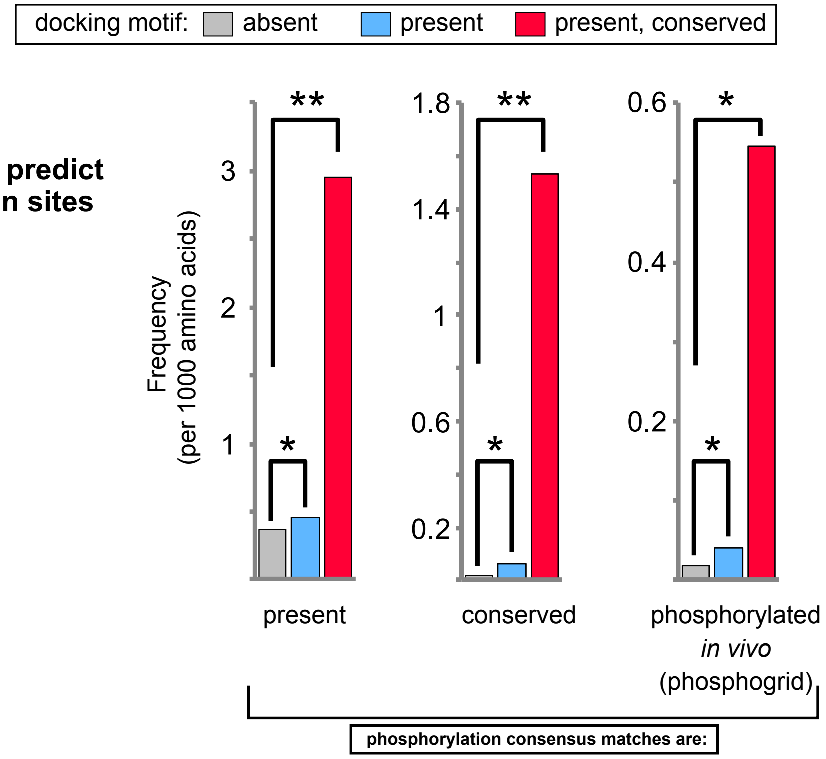

B.

phosphorylation consensus in proteins with docking motifs are more likely to be conserved and phosphorylated in vivo.

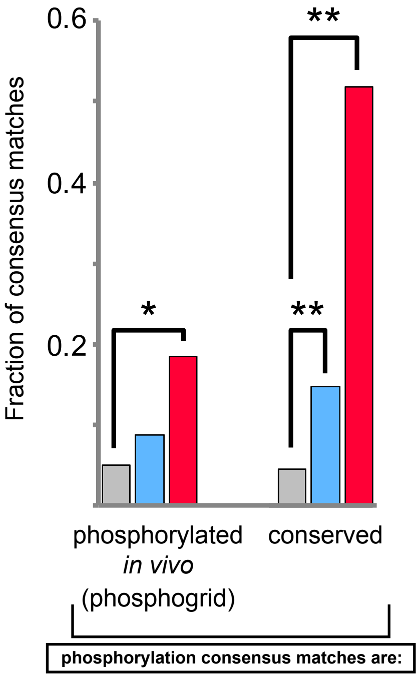

* $p < 0.05$

** $p < 10^{-5}$
