# Supplementary material for: The Structure of an NDR/LATS Kinase–Mob Complex Reveals a Novel Kinase–Coactivator System and Substrate Docking Mechanism
Source: PLoS Biol. 2015 May 12;13(5):e1002146. doi: 10.1371/journal.pbio.1002146 (PMC4428629; doi:10.1371/journal.pbio.1002146)
Supplement: S1 Table — (DOCX) [file pbio.1002146.s016.docx]

**S10** **Table** Data collection and refinement statistics

|  | Cbk1(D475A/T743E)–  Mob2 (crystal form A) | Cbk1(D475A/T743E)–  Mob2 (crystal form B) | Cbk1(D475A)–  Mob2 |
| --- | --- | --- | --- |
| **Data collection** |  |  |  |
| Space group | P 4_1_ 2_1_ 2 | C 1 2 1 | C 1 2 1 |
| Cell dimensions |  |  |  |
| *a*, *b*, *c* (Å) | 129.38, 129.38, 231.69 | 79.74, 139.06, 187.58 | 138.43, 79.98, 117.58 |
| *α*, *β*, *γ* (°) | 90, 90, 90 | 90, 90.1, 90 | 90, 117.6, 90 |
| Resolution (Å) | 49.59-4.5 (4.66-4.5)* | 46.49-3.6 (3.69-3.6) | 47.34-3.3 (3.39-3.3) |
| *R*_merge_ | 4.9 (67) | 6.4 (45) | 8.8 (68) |
| *I*/σ*I* | 23.26 (2.83) | 14.60 (2.52) | 13.86 (2.26) |
| Completeness (%) | 99.97 (100) | 98.8 (91.5) | 99.4 (99.8) |
| Redundancy | 4.3 (4.3) | 3.4 (3.0) | 3.4 (3.1) |
|  |  |  |  |
| **Refinement** |  |  |  |
| Resolution (Å) | 49.59-4.5 | 46.49-3.6 | 47.34-3.3 |
| No. reflections | 12260 | 23527 | 17276 |
| *R*_work/_ *R*_free_ | 0.2876/0.3104 | 0.2580/0.3194 | 0.2704/0.3068 |
| No. atoms |  |  |  |
| Protein | 4100 | 7467 | 4030 |
| Ligand/ion | 31 | 31 | 31 |
| Water | 0 | 0 | 0 |
| B-factors |  |  |  |
| Protein | 149.1 | 111.1 | 104.5 |
| Ligand | 169.5 | 61.0 | 93.3 |
| Water | - | - | - |
| R.m.s deviations |  |  |  |
| Bond lengths (Å) | 0.007 | 0.011 | 0.011 |
| Bond angles (º) | 1.73 | 1.87 | 1.86 |
| PDB ID | 4LQP | 4LQQ | 4LQS |

Data were collected on single crystals.

*Highest resolution shell is shown in parenthesis.
